# Reprogramming of the LXRα Transcriptome Sustains Macrophage Secondary Inflammatory Responses

**DOI:** 10.1002/advs.202307201

**Published:** 2024-03-28

**Authors:** Juan Vladimir de la Rosa, Carlos Tabraue, Zhiqiang Huang, Marta C. Orizaola, Patricia Martin‐Rodríguez, Knut R. Steffensen, Juan Manuel Zapata, Lisardo Boscá, Peter Tontonoz, Susana Alemany, Eckardt Treuter, Antonio Castrillo

**Affiliations:** ^1^ Unidad de Biomedicina (Unidad Asociada al CSIC) Instituto Universitario de Investigaciones Biomédicas y Sanitarias (IUIBS) de la Universidad de Las Palmas de Gran Canaria Las Palmas 35016 Spain; ^2^ Departamento de Morfología Universidad de Las Palmas de Gran Canaria Las Palmas 35016 Spain; ^3^ Department of Biosciences and Nutrition Karolinska Institutet, NEO Huddinge 14183 Sweden; ^4^ Center for Translational Medicine and Jiangsu Key Laboratory of Molecular Medicine Medical School Nanjing University Nanjing 210093 P. R. China; ^5^ Department of Metabolic and Immune Diseases. Instituto de Investigaciones Biomédicas Sols‐Morreale Centro Mixto Consejo Superior de Investigaciones Científicas CSIC‐Universidad Autónoma de Madrid Madrid 28029 Spain; ^6^ Division of Clinical Chemistry, Department of Laboratory Medicine Karolinska Institute Huddinge 14186 Sweden; ^7^ Centro de Investigación Biomedica en Red sobre Enfermedades Cardiovasculares (CIBERCV) Madrid 28029 Spain; ^8^ Department of Pathology and Laboratory Medicine University of California Los Angeles UCLA California 90095 USA

**Keywords:** gene expression, inflammation, macrophage, nuclear receptor LXR

## Abstract

Macrophages regulate essential aspects of innate immunity against pathogens. In response to microbial components, macrophages activate primary and secondary inflammatory gene programs crucial for host defense. The liver X receptors (LXRα, LXRβ) are ligand‐dependent nuclear receptors that direct gene expression important for cholesterol metabolism and inflammation, but little is known about the individual roles of LXRα and LXRβ in antimicrobial responses. Here, the results demonstrate that induction of LXRα transcription by prolonged exposure to lipopolysaccharide (LPS) supports inflammatory gene expression in macrophages. LXRα transcription is induced by NF‐κB and type‐I interferon downstream of TLR4 activation. Moreover, LPS triggers a reprogramming of the LXRα cistrome that promotes cytokine and chemokine gene expression through direct LXRα binding to DNA consensus sequences within cis‐regulatory regions including enhancers. LXRα‐deficient macrophages present fewer binding of p65 NF‐κB and reduced histone H3K27 acetylation at enhancers of secondary inflammatory response genes. Mice lacking LXRα in the hematopoietic compartment show impaired responses to bacterial endotoxin in peritonitis models, exhibiting reduced neutrophil infiltration and decreased expansion and inflammatory activation of recruited F4/80^lo^‐MHC‐II^hi^ peritoneal macrophages. Together, these results uncover a previously unrecognized function for LXRα‐dependent transcriptional cis‐activation of secondary inflammatory gene expression in macrophages and the host response to microbial ligands.

## Graphical abstract

The inflammatory response is a defense mechanism of the innate immune system against infections and other injuries. In response to microbial components, macrophages activate primary and secondary inflammatory gene programs crucial for host defense. De la Rosa et al. provide evidence that inflammation triggers a reprogramming of the transcription factor liver X receptor α (LXRα), that sustains cytokine and chemokine gene expression through direct binding to DNA consensus sequences and collaboration with NF‐κB at enhancers of secondary inflammatory response genes. This novel TLR4‐LXRα‐NF‐κB axis supports macrophage inflammatory gene expression and in vivo immune‐cell recruitment during inflammatory responses to microbial ligands, positioning LXRα as a key factor in the transcriptional regulation in the late stages of inflammation.

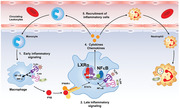

## 1 Introduction

The inflammatory response is a defense mechanism of the innate immune system against infections and other injuries. Inflammation is rapidly initiated aiming to isolate and eliminate the damaging stimuli, leading to tissue repair and healing to restore homeostasis.^[^ ^1^ ^]^ Thus, an effective inflammatory response must be robust enough, but temporarily restricted until the injury has been eliminated. Although inflammation is a crucial protective reaction, uncontrolled chronic inflammation can cause or aggravate several life‐threatening diseases, including cancer, diabetes or cardiovascular diseases.^[^ ^2^, ^3^ ^]^

Macrophages are phagocytic cells that are present in all tissues and orchestrate decisive steps at all stages of inflammation. In response to infection, macrophages detect pathogen molecular structures by innate sensors, such as Toll‐like receptors (TLR) that recognize lipopeptides, single‐stranded DNA or double‐stranded RNA.^[^ ^4^ ^]^ Engagement of TLRs and the adapter proteins Myeloid differentiation primary response 88 (MyD88) and Toll/Interleukin‐1 receptor domain‐containing adapter protein inducing Interferon beta (TRIF) leads to activation of NF‐κB, AP‐1, and IRF transcription factors (so‐called ´Signal‐Dependent Transcription Factors´, SDTFs), which rapidly promote the expression of many cytokines and soluble factors, such as interleukins and interferons.^[^ ^5^ ^]^ This primary set of autocrine and paracrine signaling proteins include type I interferons (IFNα/β), which in turn promote the expression of many secondary‐response genes,^[^ ^5^, ^6^ ^]^ including additional waves of cytokines and genes encoding for enzymes that produce reactive species important for pathogen killing.^[^ ^5^, ^7^, ^8^ ^]^ Upon activation, binding of SDTFs to cis‐regulatory elements within enhancer regions of inflammatory genes is facilitated through open chromatin regions, that are maintained accessible by master macrophage regulators (known as ´Lineage‐Determining Transcription Factors´, LDTFs), such as PU.1, C/EBP or IRF8^[^ ^7^ ^]^ and chromatin remodeling complexes.^[^ ^9^, ^10^, ^11^, ^12^ ^]^ Thus, the transcriptional response of macrophages to microbial components is accomplished by sequential waves of gene induction controlled by different combinations of SDTFs and other transcriptional regulators.

The liver X receptors (LXRα, encoded by the gene *Nr1h3* and LXRβ, encoded by the gene *Nr1h2*) are members of the nuclear receptor superfamily of transcription factors that play crucial roles in sterol homeostasis in mammals, and also regulate inflammation and immunity.^[^ ^13^ ^]^ LXRs operate as obligate heterodimers with the retinoid X receptors and their endogenous ligands include various intermediates of the cholesterol biosynthetic pathway.^[^ ^10^ ^]^ Much of the LXR pharmacology and target‐gene discovery, however, has been studied with potent, non‐steroid synthetic ligands.^[^ ^14^ ^]^ Mechanistically, LXRs positively regulate gene expression through direct binding to cognate DNA response elements (LXREs) within cis‐regulatory regions (i.e., enhancers and promoters) of target genes.^[^ ^14^, ^15^ ^]^ In addition, LXR‐dependent gene repression has been described through several mechanisms, especially in inflammatory macrophages.^[^ ^16^, ^17^, ^18^ ^]^ Attenuation of inflammation is observed when synthetic LXR ligands are administered before the injury, as reported in cellular and in vivo models of inflammation.^[^ ^16^, ^17^, ^19^ ^]^ Restraining inflammation could be beneficial in certain settings, but may also weaken the host defense against infections, as shown by synthetic LXR ligands during pulmonary infection.^[^ ^20^, ^21^ ^]^ Moreover, alleviation of inflammation exerted by synthetic LXR ligands contrasts with the protection against pathogens demonstrated by endogenous LXR activity in vivo in infection models. LXR‐deficient mice present defective innate immunity against *Listeria monocytogenes* ^[^ ^22^ ^]^ or *Mycobacterium tuberculosis* ^[^ ^23^ ^]^ and an inadequate response to cecal ligation and puncture model of polymicrobial sepsis,^[^ ^24^ ^]^ suggesting that endogenous LXR activity, instead of repressing, potentiates innate immune responses.

LXR‐dependent immune protection against bacterial infection has been shown to be mediated mainly by LXRα.^[^ ^22^, ^23^ ^]^ However, the molecular connections between innate immune pathways and endogenous LXRα activity in macrophages have not been explored in depth. The objective of this study was to investigate the transcriptional regulation, differential DNA binding and in vivo activity of LXRα in response to a prototypical microbial component, the TLR4 agonist LPS. Using primary macrophages with genetic inactivation of key inflammatory signaling components, transcriptional profiling and ChIP‐seq studies, we demonstrate that TLR4 signaling induced a secondary inflammatory response that involves LXRα transcriptional induction, which in turn rewires its genomic binding landscape to cis‐regulatory regions of inflammatory genes, whereby it promotes cytokine and chemokine gene expression. These data uncovered a novel TLR4‐LXRα axis that sustains macrophage inflammatory gene expression and in vivo immune‐cell recruitment during inflammatory responses to microbial ligands.

## 2 Results

### 2.1 Microbial Components Induce LXRα Transcription during Secondary Inflammatory Responses

**Figure 1:**
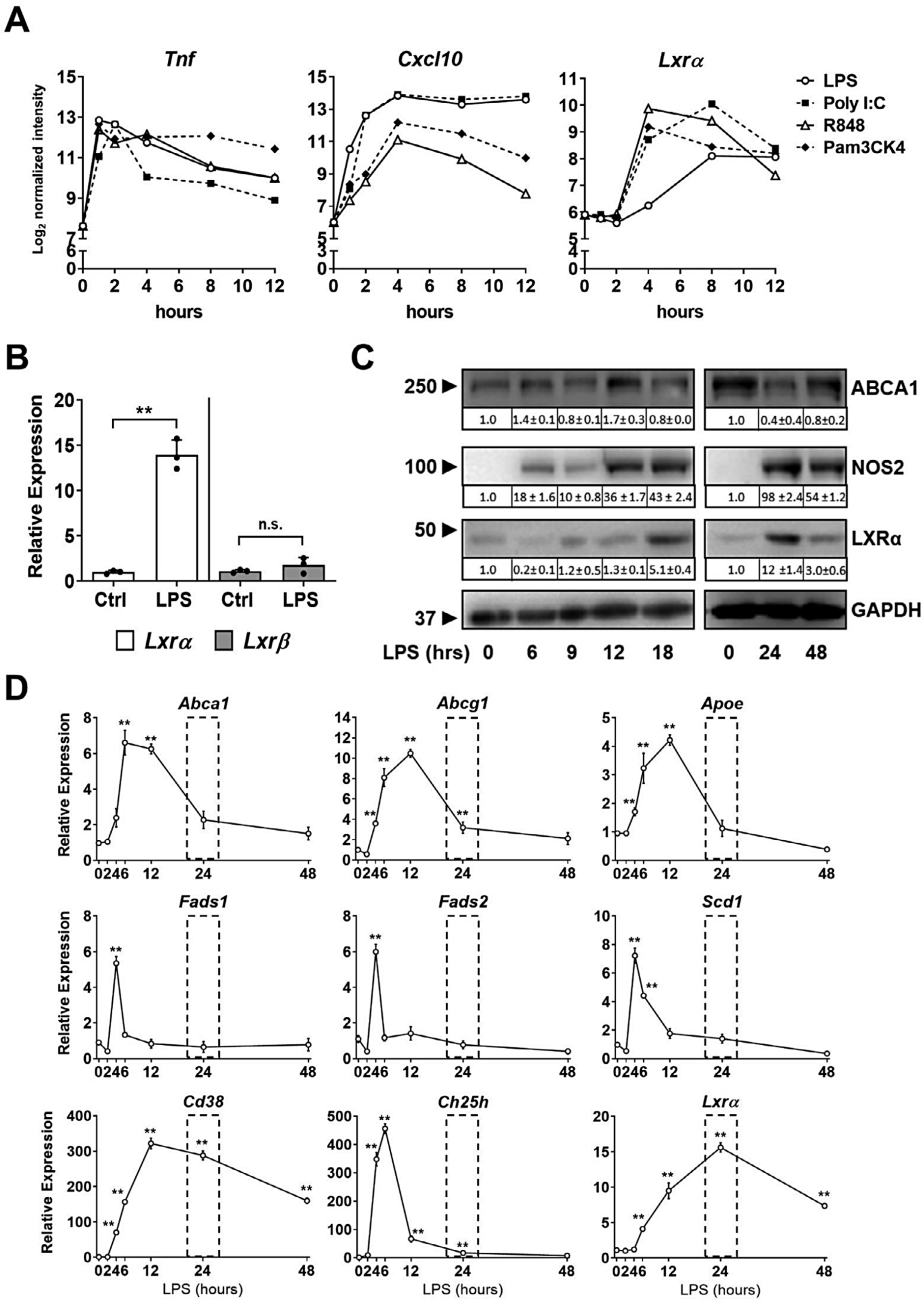
A) Time‐course of mRNA expression of the indicated genes in response to TLR agonists: LPS (**TLR4**), Poly I:C (**TLR3**), PAM3CSK4 (**TLR2**) and R848 (**TLR7** and **TLR8**); normalized values of RNA expression were obtained from database ArrayExpress E‐TABM‐310.^[^ ^30^ ^]^ B) Relative mRNA expression of *Lxrα* and *Lxrβ* in BMDMs cultured 24 h with LPS (100 ng ml^−1^). C) Protein levels of LXRα, ABCA1 and NOS2 in WT BMDMs cultured with LPS (100 ng ml^−1^) or PBS for different times (0 – 48 h). Band intensities quantified by densitometry are displayed below each blot image. D) Relative mRNA expression levels (0 – 48 h.) for indicated genes in WT BMDMs cultured with LPS (100 ng ml^−1^). Expression data in B–D) are represented as mean ± SD from *n* = 3 experiments. Significant differences between mean values with the control condition were indicated (* *p* < 0.05 and ** *p* < 0.01).

Previous work from our group and others demonstrated that macrophage LXRs participate in the regulation of inflammation and immunity,^[^ ^16^, ^17^, ^18^ ^]^ but the impact of microbial ligands and their signaling pathways on endogenous LXR transcriptional activity has not been explored in depth. Also, in a former study using ectopic expression of LXRα or LXRβ in an LXR‐deficient background macrophage model, we demonstrated non‐overlapping transcriptional actions of each receptor beyond fatty acid and sterol metabolism.^[^ ^25^ ^]^ Since LXRα plays a singular role in the protection against infections,^[^ ^22^, ^23^ ^]^ we focused our interest on the regulation of *Nr1h3* gene transcription (for clarity with the nomenclature, we will use *Lxrα* for the gene/mRNA and LXRα for the protein) and LXRα protein expression in macrophages activated by microbial ligands. We chose bone marrow‐derived macrophages (BMDM) differentiated with MCSF as cell model.^[^ ^25^ ^]^ Although earlier reports showed increased *Lxrα* mRNA expression in LPS‐activated myeloid cells,^[^ ^26^, ^27^, ^28^, ^29^ ^]^ the molecular mechanisms underlying this induction and the identification of LXRα downstream targets in the context of inflammation, have not been thoroughly investigated. To establish a time frame in which *Lxrα* mRNA was induced by TLR agonists in BMDM, compared to other prototypical primary and secondary‐responsive genes, we investigated the expression of *Tnf*, *Cxcl10, Nos2* and *Lxrα* genes from public datasets.^[^ ^30^ ^]^ Activation of TLR2, TLR3, TLR4 and TLR7/8 increased the expression of early and late inflammatory genes with different timing, as expected (**Figure** **1**A). While *Tnf* expression acutely raised during the first hour post‐challenge, TLR‐dependent expression of *Lxrα* augmented several hours later, similar to other secondary‐responsive genes, such as *Nos2* (Figure 1A; Figure S1A, Supporting Information). Our experiments reproducibly showed maximal induction of *Lxrα* mRNA at 18–24 h post LPS, whereas *Lxrβ* expression did not change substantially (Figure 1B).

LXRα protein levels gradually increased in LPS‐stimulated macrophages, similar to NOS‐2 (Figure 1C). LPS induction of *Lxrα* mRNA showed LPS dose‐dependency and changes in *Lxrα* mRNA or nascent hnRNA were abolished in TLR4 ‐/‐ macrophages (Figure S1B and C, Supporting Information). The transcription inhibitor Actinomycin D declined both *Lxrα* mRNA and nascent hnRNA after the LPS challenge, consistent with the notion that TLR4 signaling directly impacts *Lxrα* primary transcription and not mRNA stability (Figure S1D, Supporting Information). Moreover, experiments employing protein synthesis inhibitor cycloheximide for different times decreased the LXRα protein levels that were induced by LPS to a similar ratio as control cells (Figure S1E, Supporting Information). This implies that the amount of accumulated LXRα protein in response to prolonged periods of TLR4 stimulation was not due to protein stabilization. Surprisingly, although *Lxrα* mRNA and protein levels potently augmented in response to LPS, the expression of classic LXR target genes did not parallel this temporal pattern. Indeed, the expression of many LXR targets, with the exception of *Cd38*, increased at early times in response to LPS, and returned to control levels or decreased at the time LXRα protein was maximal (Figure 1C,D). As previously reported,^[^ ^31^ ^]^ we confirmed that the expression of the cholesterol 25 hydroxylase (*Ch25h*, which catalyzes the conversion of cholesterol into 25‐Hydroxycholesterol, 25‐HC) was also potently induced by LPS, and would be a potential source of the oxysterol ligand 25‐HC^[^ ^32^ ^]^ during inflammation (see below, also connected to Figure 4; Figure S3, Supporting Information). Together, these experiments revealed that LXRα expression, but not LXRβ, was induced during extended phases of the inflammatory response in macrophages but its endogenous activity was not linked to positive regulation of established LXR target genes.

### 2.2 Intracellular Determinants Responsible for LXRα Expression in Inflammatory Macrophages

**Figure 2:**
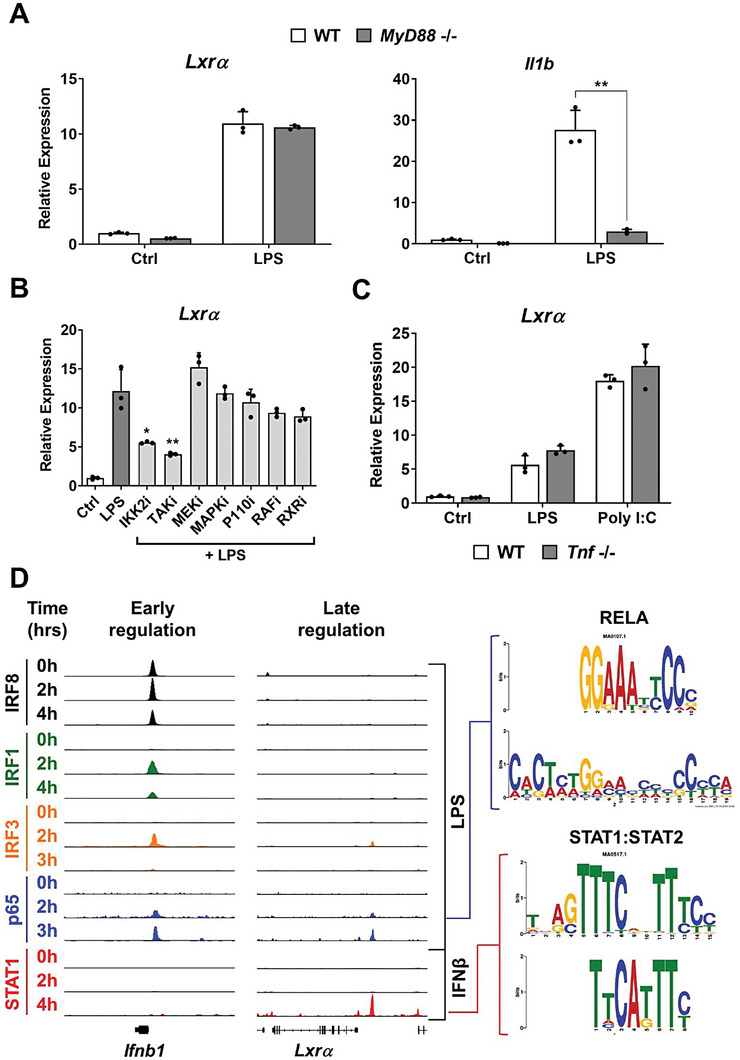
A) Relative mRNA expression levels of *Lxrα* and *Il1b* in BMDMs from WT and MyD88‐/‐ mice cultured with LPS (100 ng ml^−1^) for 24 h. B) Relative mRNA expression of *Lxrα* in BMDMs cultured 24 h with LPS alone (100 ng ml^−1^) or with inhibitors BI605906 (IKK2, 1 µM), 5Z‐7‐oxozeanol (TAK1, 1 µM), PD0325901 (MEKi 10 µM), PD98059 (MAPKi 10 µM), PIK‐75‐hydrocloruro (P110αi 2 µM), SB590885 (RAFi 1 µM), HX531 (RXRi 2 µM). C) Relative mRNA expression levels of *Lxrα* in WT and *Tnf* ‐/‐ BMDMs cultured 24 h with LPS (100 ng ml^−1^) or Poly I:C (10 ug ml^−1^). D) IGV genome browser ChIP‐seq data for IRF8, IRF1, IRF3, p65 and STAT1 transcription factors in the vicinity of *Ifn1b* and *Lxrα* loci at 0, 2 and 4 h of LPS or IFNβ treatment, and RELA NF‐κB and STAT1 consensus sequence motifs are depicted (right). All mRNA expression data were represented as mean ± SD from two or three experiments. Significant differences between mean values of LPS WT versus MyD88‐/‐ (in A) and LPS versus LPS+inhibitors (in B) were indicated (* *p* < 0.05 and ** *p* < 0.01).

The observation that *Lxrα* expression was induced by several TLR agonists led us to investigate common intracellular pathways downstream of TLRs that might be responsible for this activation. First, we examined the signaling downstream MyD88, a common adaptor protein crucial for most TLR‐dependent signals. Analysis of gene expression using MyD88‐/‐ macrophages revealed that, while *Il1b* expression was blunted, induction of *Lxrα* was similar in WT and MyD88‐/‐ macrophages (**Figure** **2**A). To refine the search for intracellular transduction signals that trigger *Lxrα* expression downstream of TLRs, we analyzed the effect of a battery of validated inhibitors that target key signaling molecules. Blocking the activation of IKKβ or TAK1 decreased *Lxrα* expression in response to LPS (Figure 2B), indicating that activators of the NF‐κB pathway participated in this regulation. Secondary inflammatory responses require autocrine activation by cytokines, such as TNFα, which in turn directly activates the NF‐κB pathway.^[^ ^33^ ^]^ We employed TNFα‐deficient macrophages to explore this possibility. The analysis revealed similar expression of *Lxrα* in both WT and *Tnf*‐/‐ macrophages in response to LPS or Poly I:C, indicating that endogenous TNFα was not required for the late induction of LXRα (Figure 2C).

To explore the potential SDTFs that could be participating in the control of LXRα expression in response to inflammatory signaling, we compared the recruitment of key SDTFs to promoter regions of early (*Infb1*) or late (*Lxrα*) response genes. Analysis of public ChIP‐seq datasets^[^ ^11^, ^12^, ^34^, ^35^ ^]^ revealed that *Infb1* promoter, but not *Lxrα*, was pre‐marked with potent IRF‐8 binding. In addition, IRF‐1, IRF‐3 and p65 NF‐κB were recruited earlier to the *Infb1* promoter in response to TLR4 signaling, whereas binding of p65 NF‐κB, but not IRFs, was observed later in response to LPS within the *Lxrα* enhancer region (Figure 2D). When comparing the genomic recruitment of SDTFs in response to INFβ stimulation, to mimic autocrine secondary response to type I IFNs, we did not observe STAT1 binding to the *Ifnb1* promoter, but prominent recruitment of p65 NF‐κB and STAT1 at the *Lxrα* enhancer region. In addition, canonical RELA NF‐κB and STAT binding motifs were found at the *Lxrα* regulatory region (Figure 2D, right). These results suggest that NF‐κB and STATs are important for the secondary induction of *Lxrα* expression by TLR signaling.

### 2.3 Inducible LXRα Expression Requires TRIF/TBK‐1/IRF‐3 Activation and Type‐I Interferons

**Figure 3:**
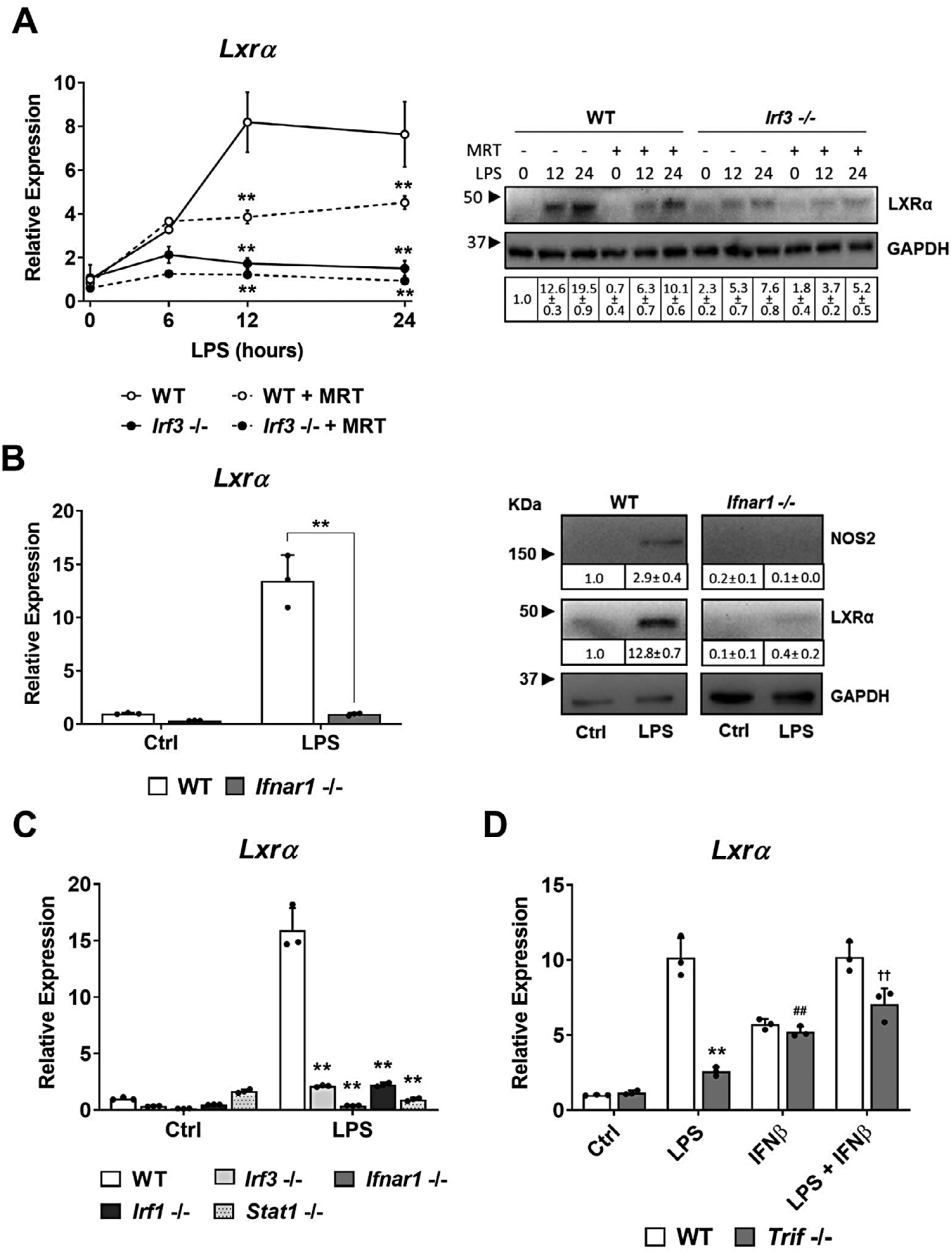
A) Left panel. Relative mRNA expression of *Lxrα* in BMDMs from WT or *Irf3*‐/‐ mice cultured with LPS (100 ng ml^−1^ for 0 – 24 h.) alone or combined with MRT67307 (TBK1 inhibitor, 2 µM), and protein levels (A, right panel) of LXRα and GAPDH from cells under the same conditions as in A; relative band intensities were quantified by densitometry. B) Left panel. Relative mRNA expression of *Lxrα* in WT or *Ifnar*‐/‐ BMDMs cultured with LPS (100 ng ml^−1^) for 24 h, and protein levels (right panel)of LXRα, NOS‐2 and GAPDH; relative band intensities were quantified by densitometry. C) Relative mRNA expression of *Lxrα* in WT or *Ifnar*‐/‐, *Stat1*‐/‐, *Irf3*‐/‐ and *Irf1*‐/‐ BMDMs cultured with LPS (100 ng ml^−1^) for 24 h. D) Relative mRNA expression of *Lxrα* in WT or *Trif*‐/‐ BMDMs cultured with LPS (100 ng ml^−1^) or IFNβ (500 U ml^−1^) alone or combined. All mRNA expression data were represented as mean ± SD from three experiments. Asterisks indicate significance between with WT and knockout cells B–D). Hashes and crosses indicate significant differences in *Trif*‐/‐ BMDM treated with IFNβ or LPS+ IFNβ compared to *Trif*‐/‐ BMDM treated with LPS alone. Significant differences between means (**, ## or †† p < 0.01).

Given the possible involvement of type I IFNs in the temporal induction of *Lxrα* expression by LPS, we studied its regulation by the TANK‐binding kinase 1 (TBK‐1) and Interferon regulatory factor 3 (IRF‐3) pathway, which is crucial for autocrine and paracrine IFN signaling.^[^ ^8^, ^36^ ^]^ *Lxrα* mRNA expression was impaired in *Irf3*‐/‐ BMDM compared to WT cells in response to LPS (**Figure** **3**A, left panel). In addition, macrophages cultured with TBK1 inhibitor MRT67307^[^ ^36^, ^37^ ^]^ partially blocked *Lxrα* induction in WT cells and had little effect in *Irf3*‐/‐ cells. The analysis of LXRα expression revealed that, although LPS‐induced LXRα levels were mostly dependent on IRF‐3 expression, there was some remaining LXRα expression in *Irf3*‐/‐ macrophages, suggesting that additional TFs were involved in the regulation of LXRα protein expression (Figure 3A, right panel). To directly address the role of type I IFNs in LXRα induction, we analyzed LXRα expression in response to LPS by real‐time qPCR and western blot in macrophages deficient in type I IFN receptor, IFNAR‐1 (Figure 3B). This analysis revealed that *Lxrα* mRNA (Figure 3B, left panel) and protein levels (Figure 3B, right panel) were severely impaired in *Ifnar1*‐/‐ macrophages compared to WT, confirming that secondary signaling driven by type I IFNs was crucial for triggering inducible LXRα expression. Moreover, using *Irf1‐/‐, Irf3‐/‐ and Stat1‐/‐* macrophages, we corroborated that inflammatory SDTFs that are activated downstream of type I IFNs were decisive for *Lxrα* mRNA expression (Figure 3C).

Because the MyD88‐independent arm of TLR3/4 signaling requires the adaptor protein TRIF^[^ ^38^, ^39^ ^]^ (also known as TICAM‐1), for TBK‐1/IRF‐3/IFNβ activation, we analyzed *Lxrα* expression in WT and *Trif*‐/‐ macrophages. In response to LPS, expression of *Lxrα* was diminished in *Trif*‐/‐ cells (Figure 3D). Importantly, challenging cells with IFNβ alone was able to partially rescue *Lxrα* expression in *Trif*‐/‐ macrophages to similar levels as WT cells, indicating that IFNβ signaling is important for *Lxrα* expression during secondary inflammatory responses. Collectively, these findings provide evidence that IFN‐β via TLRs/TRIF/TBK1/IRF3 signaling is crucial for the inducible expression of LXRα in macrophages as a secondary‐responsive gene to microbial‐ligand challenging.

### 2.4 Global Profiling Identifies Specific Subsets of LXRα‐Dependent Genes Following TLR4 Activation

**Figure 4:**
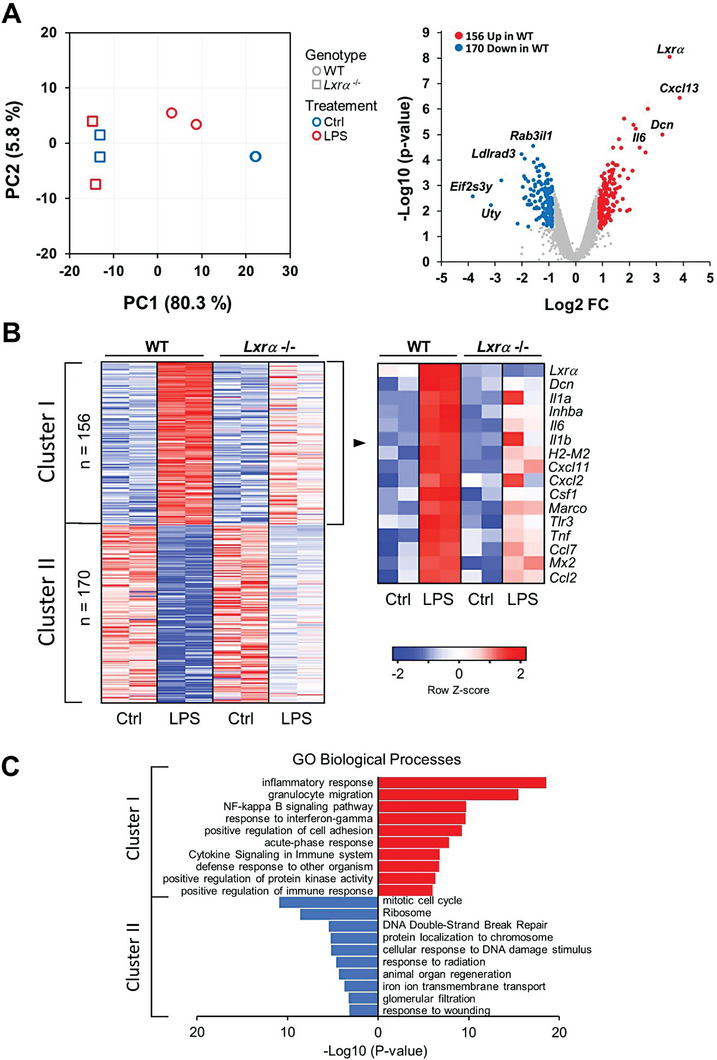
A) Principal Component Analysis (PCA) of detectable mRNAs (left graph) and volcano plot (right graph) of transcriptional profiling data comparing WT and *Lxrα* ‐/‐ BMDMs cultured with LPS (100 ng ml^−1^) for 24 h. Differentially expressed genes (p‐value < 0.05, FC > 2) are shown in red (Up‐regulated) or blue (Down‐regulated) in *Lxrα*‐/‐. Data are from two experiments with n = 2 biological replicates per group, and B) Heatmap plots showing differentially expressed genes among replicates and details of top regulated genes in Cluster I. C) Bar graph of Gene Ontology (GO) enrichment analysis for differentially expressed genes red (Down‐regulated) or blue (Up‐regulated) in *Lxrα* ‐/‐ BMDMs.

To reveal the full spectrum of endogenous LXRα activity and its downstream signaling, we performed gene expression profiling in WT and *Lxrα*‐/‐ macrophages cultured 24 h with LPS. Principal component analysis (PCA) of gene expression data showed that replicate profiles of LPS‐stimulated *Lxrα*‐/‐ macrophages differed considerably from their WT counterparts (**Figure** **4**A, left panel). Volcano‐plot and heat‐map analysis revealed clusters of genes differentially regulated (Fold Change [FC] ≥ 2 and P‐value < 0.05) in *Lxrα*‐/‐ macrophages in response to LPS. Clustering of differentially expressed genes (FC>2 across genotypes) identified two principal groups of genes (clusters I, II) (Figure 4A right panel and 4B). Cluster I comprised genes that were defectively induced by LPS in *Lxrα*‐/‐ macrophages, whereas cluster II is composed by genes that did not show similar repression in *Lxrα*‐/‐ after LPS treatment, compared to WT macrophages. Gene ontology and pathway analysis of cluster‐I revealed enrichment of inflammatory processes such as NF‐κB signaling pathway, response to interferon and acute phase response (Figure 4C). Indeed, genes that showed the greatest differences in Cluster I corresponded to hallmark proinflammatory cytokines (*Tnf, Il6, Il1b and Il1a*), and chemokines (*Ccl2, Ccl7, Cxcl2 and Cxcl11*) (Figure 4B, right panel) that were more expressed in WT compared to *Lxrα*‐/‐ macrophages in response to LPS. In contrast, cluster II contained transcripts more expressed in *Lxrα*‐/‐ macrophages and showed enrichment in pathways related to cell‐cycle, cellular response to DNA damage and response to wounding (Figure 4B,C). Moreover, we compared the mRNA expression of a panel of selected cytokines, chemokines or anti‐microbial genes in WT, *Lxrα*‐/‐ and *Lxrβ*‐/‐ BMDM in response to increasing times of LPS stimulation (Figure S2, Supporting Information). The analysis of qPCR data showed that expression of *Il1a, Il1b, Il6, Inhba, Marco and Tnf* was decreased in *Lxrα*‐/‐ macrophages at prolonged, but not at short times after LPS challenge. Expression of other inflammatory genes, such as *Nos2* or *Mx1* did not significantly depend on *Lxrα* or *Lxrβ* expression after LPS treatment (Figure S2, Supporting Information).

The observation that several inflammatory genes required *Lxrα* expression for their full induction at later, but not early times of LPS stimulation was intriguing. In addition, our previous data presented in Figure 1d showed that early LPS stimulation induced the expression of targets involved in cholesterol or fatty acid metabolism, yet uncoupled to the temporal LXRα maximal induction. These facts questioned if perhaps LPS regulation of established LXR targets required both *Lxrα* and *Lxrβ* for their expression and whether the oxysterol 25‐HC (produced by the prominent induction of *Ch25h*, Figure 1D and reported before^[^ ^31^ ^]^) would serve as endogenous LXR ligand^[^ ^40^ ^]^ for the early and late transcriptional actions of LPS signaling. Analyzing independent data from available expression profiling of LPS‐stimulated BMDM,^[^ ^41^ ^]^ obtained from either WT, *Lxrα,β‐/‐* or *Ch25h*‐/‐ mice, showed that expression of most LXR targets was higher in *Ch25h*‐/‐ in response to LPS (with the exception of *Abca1* and *Abcg1*), but required *Lxrα* and *LXRβ* for their full expression (Figure S3, Supporting Information). Interestingly, many of the pro‐inflammatory genes identified from Cluster I of our profiling (Figure 4B, which were improperly induced by LPS in *Lxrα*‐/‐ macrophages) were also downregulated in *Lxrα,β‐/‐*, thus reinforcing our conclusions (Figure S3, Supporting Information). In addition, expression of some of these pro‐inflammatory genes, including *Inhba, Il6, Ccl2 and Ccl7* was partially downregulated in *Ch25h*‐/‐, suggesting that 25‐HC might be involved as endogenous ligand targeting LXRα for their transcriptional activation. Expression of *Il1a, Il1b or Cxcl2*, however, was upregulated in *Ch25h*‐/‐, indicating that other ligand/s besides 25‐HC might be participating as LXRα ligands for their regulation. Overall, these results suggested, surprisingly, that the inducible transcription of endogenous LXRα at later stages of macrophage responses was not playing an anti‐inflammatory role, but rather was important for the expression of a battery of secondary‐responsive pro‐inflammatory genes.

### 2.5 Deciphering the LXRα Genomic Binding Landscape in Macrophages in Response to LPS

**Figure 5:**
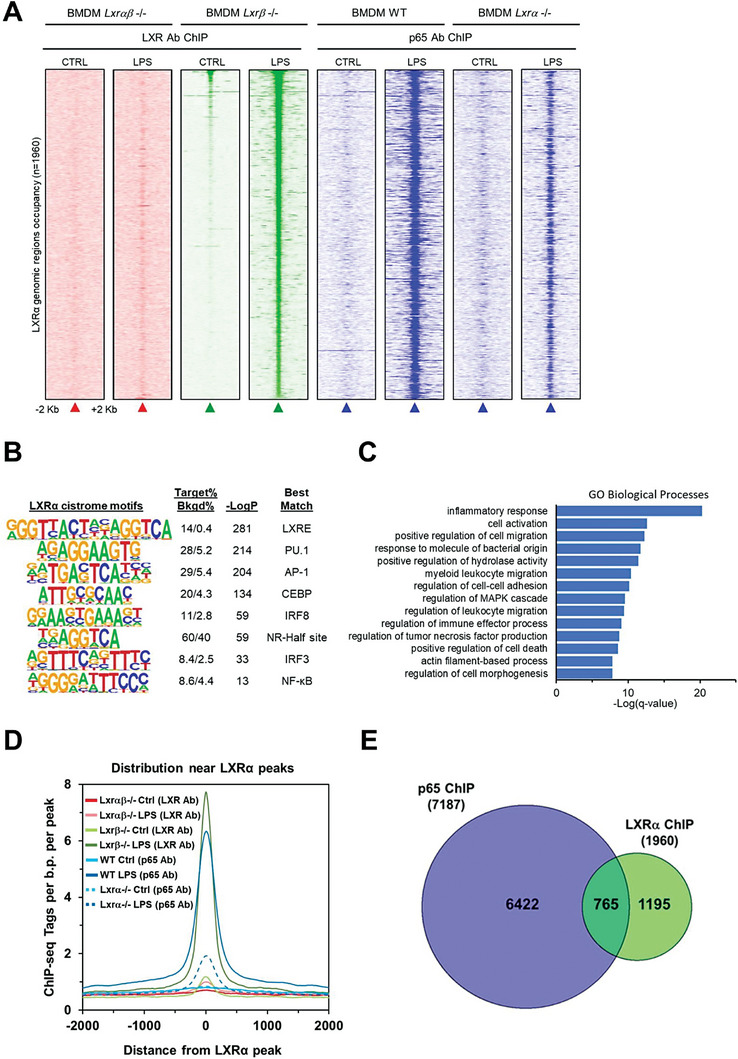
A) Heatmap representation of normalized tag densities obtained through LXRα ChIP‐seq around 2 Kb of *de novo* LXRα binding locations in *Lxr*β‐/‐ BMDMs cultured with or without LPS for 24 h (density map represented in green). As negative control, ChIP‐seq was performed in *Lxr*α,β‐/‐ BMDM (left panels, in red). Parallel heatmap representation of p65 NF‐κB ChIP‐seq in WT and *Lxr*α‐/‐ BMDMs cultured with or without LPS for 24 h (right panels, in blue). B) Sequence motif analysis associated with *de novo* LXRα peaks in LXRβ‐/‐ BMDMs cultured with or without LPS for 24 h. C) Gene Ontology (**GO**) analysis associated with *de novo* LXRα peaks. D) Distribution of ChIP‐seq tags surrounding the LXRα peak centers. E) Venn‐diagram representation of total number of LXRα and p65 NF‐κB peaks obtained by ChIP‐seq analysis of BMDM challenged with LPS for 24 h.

Previous studies reported that TLR ligands induce partial reprogramming of macrophage SDTFs at predefined genomic cis‐regulatory locations.^[^ ^42^ ^]^ To study whether LXRα was potentiating secondary inflammation through direct binding to genomic regulatory regions of target genes, we optimized an LXRα ChIP‐seq assay using an LXR‐dual polyclonal antibody^[^ ^43^ ^]^ (Figure S4, Supporting Information) and *Lxrβ‐/‐* macrophages that express LXRα and showed differential regulation of inflammatory gene expression when compared to *Lxrα‐/‐* cells at 24 h post LPS challenge (Figure S2, Supporting Information). We searched for *de novo* LXRα binding sites induced by LPS compared to control, using *Lxrα,β‐/‐* double deficient macrophages as negative control. Bioinformatic analysis revealed a dynamic occupation of LXRα binding sites (1960 genomic regions) in response to late LPS signaling (**Figure** **5**A). Analysis revealed that secondary response to LPS induced robust LXRα binding at sites in which LXRα was very little present or not present in unstimulated cells, suggesting that a prolonged inflammatory response promoted a novel LXRα binding landscape distinct from the vicinity of classic, sterol metabolic LXR target genes (Figure 5A; Figure S5A, Supporting Information). Indeed, LXRα binding at classic target‐gene loci did not change significantly after LPS stimulation (Figure S5A, Supporting Information, left panel) and suggested that newly synthesized LXRα might act as an inflammatory SDTF in combination with other transcription factors.

To gain insight into additional transcription factors that might bind to those regulatory regions in combination with LXRα, we studied the sequence patterns enriched within LXRα‐binding sites by motif analysis (Figure 5B). This revealed that LXRα preferentially binds regions enriched for the canonical LXR binding site (LXRE) or modified LXR sites containing a half‐site nuclear receptor binding motif (AGGTCA), along with binding sites for the master macrophage LDTF PU.1 and inflammatory SDTFs including ATF/AP1 family members, NF‐κB and IRFs (Figure 5B). Analysis of gene ontology and biological pathways of LXRα genomic‐binding sites revealed enrichment of the inflammatory response and myeloid leukocyte migration (Figure 5C). We reasoned that LPS‐inducible, chromatin‐bound LXRα, might help promoting late inflammatory gene expression through collaboration with other SDTFs. We focused on NF‐κB as this TF appeared enriched in both, the GO biological pathways of Cluster I of genes defectively induced by LPS in *Lxrα*‐/‐ macrophages (Figure 4C), and the motif analysis of adjacent sequences to those were newly synthesized LXRα was deposited (Figure 5B). To test the possible influence of LXRα on NF‐κB recruitment at cis‐regulatory regions of inflammatory genes, we performed ChIP‐seq analysis of the p65 component of NF‐κB in WT and *Lxrα*‐/‐ macrophages at early (3 h) and late (24 h) stimulation with LPS (Figure 5A,D,E; Figures S5 and S6, Supporting Information). Long treatment for 24 h induced a large number of accessible chromatin regions with p65 binding (total 7187 peaks). When we looked at the p65 binding peaks that were aligned with the LXRα‐sensitive regions, we observed that an important proportion of p65 binding had decreased in *Lxrα*‐/‐ macrophages as compared with WT macrophages at late LPS times (Figure 5E; 765 out of 1960). This indicates that ≈40% of the regions co‐occupied by LXRα and p65 in WT cells in response to LPS experienced an important reduction of NF‐κB binding in *Lxrα*‐/‐ macrophages (Figure 5A,D). Examples of genomic loci which showed inducible LXRα binding in response to LPS that displayed a reduction of p65 binding in *Lxrα*‐/‐ macrophages include enhancer regions important for *Il1a, Il6 or Ccl2/Ccl7* expression. In contrast, those same locations presented a similar p65 binding at at 3 h post‐LPS stimulation in WT and *Lxrα*‐/‐ macrophages (Figure S6, Supporting Information).

**Figure 6:**
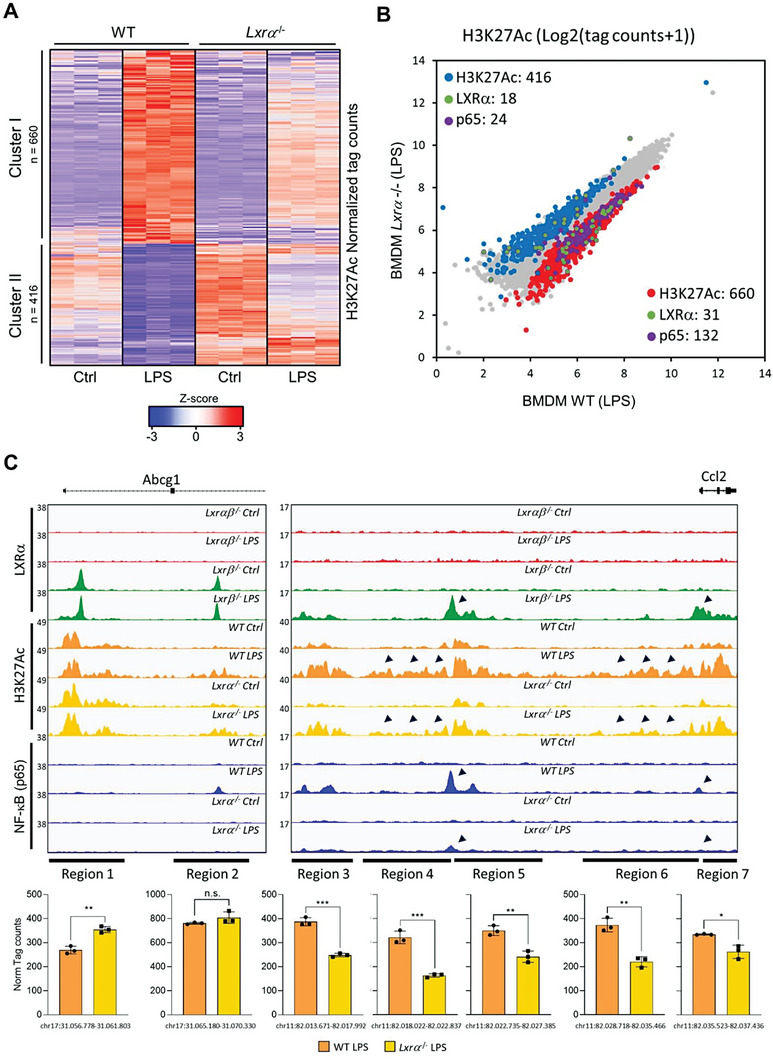
A) Heatmap representation of normalized tag densities of H3K27ac genomic regions, obtained through ChIP‐seq analysis in control versus LPS WT and *Lxr*α‐/‐ BMDM. Each row is z‐score normalized tag counts for a peak. Data from *n* = 3 biological replicates per group. Color codes indicate significant changes (p‐adj < 0.05, FC > 2) in H3K27ac with or without LPS. B) Scatterplot of discrete H3K27ac regions in WT versus *Lxr*α‐/‐ BMDM cultured with LPS (cluster regions from A), and their associated LXRα (green) or p65 NF‐κB (purple) binding sites. Data are from *n* = 3 per group. C) IGV genome browser images for the indicated genomic regions showing LXRα, p65 NF‐κB or H3K27ac peaks in the indicated BMDMs cultured with or without LPS for 24 h. Bar graphs illustrate H3K27ac normalized tag counts for the indicated genomic regions in WT and *Lxr*α‐/‐ BMDMs cultured with LPS for 24 h. Normalized tag counts data were represented as mean ± SD from three experiments. Significant differences between means values were indicated (* *p* < 0.05 and ** *p* < 0.01).

We also employed ChIP‐seq to study the H3K27Ac acetylation signal, which has been directly associated with active enhancers^[^ ^44^, ^45^ ^],^ using WT and *Lxrα*‐/‐ macrophages. As shown in **Figure** **6**A, 660 enhancer regions showed defective H3K27Ac up‐regulation in *Lxrα*‐/‐ macrophages in response to LPS. Genomic regions in this cluster included regulatory vicinities of genes coding for CCLs, CCRs and CXCLs chemokines, which are associated with functions in defense response, cytokine response and neutrophil recruitment. Given the important crosstalk of LXRα and p65 NF‐κB TFs at genomic regions that were reprogrammed after long LPS stimulation times, we aligned discrete enhancer regions that either gained or lost H3K27ac marks with those LXRα and p65 NF‐κB peaks (Figure 6B, scatter plot). Up‐regulated H3K27ac regions induced by LPS (marked in red in Figure 6B) contained more LXRα (31 vs. 18) and p65 (132 vs. 24) binding sites than regions that exhibited decreased H3K27ac marks (marked in blue in Figure 6B), consistent with the hypothesis that the co‐occurrence of LXRα and NF‐κB was important within genomic regions associated with transcriptional activation during inflammation. In addition, examples of discrete enhancer regions in the vicinity of the *Ccl2, Il1r1* and *Abcg1* loci corroborated the proposed mechanism that operates within inflammatory or non‐inflammatory genes (Figure 6C; Figure S5B, Supporting Information). A group of enhancers that control the expression of cytokines and chemokines showed greater H3K27Ac signal in response to LPS in WT BMDM, which was diminished in *Lxrα*‐/‐ macrophages, possibly due to reduced recruitment of p65 NF‐κB and other transcriptional coregulators, resulting in decreased histone acetylation and inadequate gene activation (Figure 6C, right panel and). On the other hand, classic LXR targets, such as *Mylip* or *Abcg1*, showed unaltered LXRα binding in response to LPS, low or no recruitment of p65 NF‐κB and minimal changes in H3K27Ac (Figure 6C; left panel and Figure S5A, Supporting Information left panel). Together, ChIP‐seq and transcriptional profiling data showed that LPS‐inducible LXRα preferentially binds to canonical LXRE sequences that are predetermined by macrophage LDTFs which, in combination with p65 NF‐κB recruitment and other SDTFs inflammatory transcription factors, promoted secondary inflammatory gene expression.

### 2.6 Endogenous LXRα Facilitates Immune‐Cell Recruitment at Inflammation Sites In Vivo

**Figure 7:**
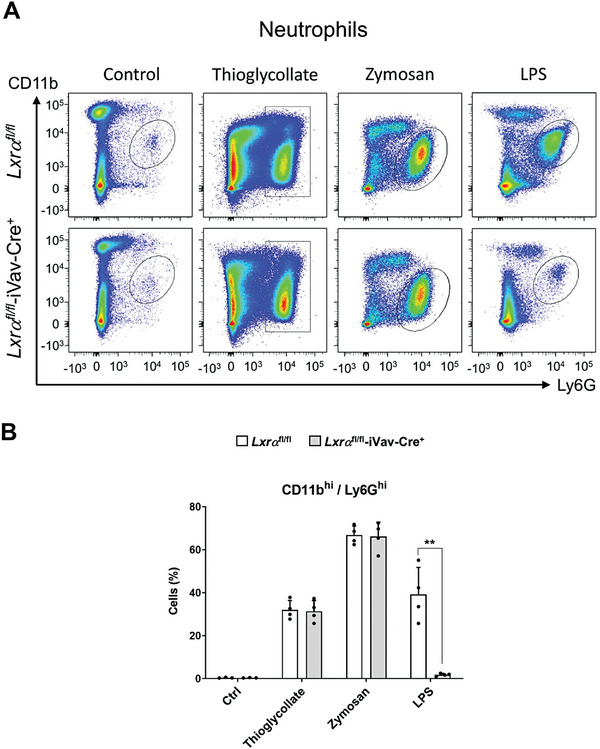
A) Flow cytometry analysis of peritoneal exudates showing Ly6G^+^/CD11b^+^ neutrophil accumulation in *Lxrα* ^fl/fl^‐iVav‐Cre^−^ (WT) and *Lxrα* ^fl/fl^‐iVav‐Cre^+^ mice injected with different stimuli: LPS (4 mg kg^−1^ per mouse), thioglycollate (2 ml of 3% solution per mouse) and zymosan (1 mg per mouse). B) Quantification of flow cytometry data showing the percentage of neutrophils among total peritoneal cells. Flow cytometry data were represented as mean ± SD from one or two experiments with *n* = 4‐5 per group. Significant differences between mean values are denoted (** *p* < 0.01).

The in vitro studies presented above indicated that LXRα participates in the maintenance of macrophage secondary inflammatory response induced by TLR stimulation. Inflammatory mediators, such as cytokines and chemokines are important for the recruitment of immune cells to sites of infection and injury.^[^ ^2^ ^]^ To investigate the role of endogenous LXRα‐dependent pathways in modulating inflammation in vivo, we used a validated model of murine peritoneal inflammation using three different challenges;^[^ ^46^, ^47^ ^]^ LPS, zymosan or thioglycollate were injected into cohorts of WT and LXRα deficient mice. To concentrate our analysis on the role of macrophages and to avoid the contribution LXRα expressed in other cells, such as hepatocytes in the liver, we employed a previously characterized C57Bl6 mouse model with LXRα deficiency in hematopoietic cells (*Lxrα* ^fl/fl^‐iVav‐Cre^+^) that showed potent recombination in macrophages in vivo.^[^ ^48^ ^]^ As a readout of the inflammation status, we used the accumulation of neutrophils in the peritoneal cavity at 24 h post‐injury.^[^ ^49^ ^]^ As expected, a prominent accumulation of neutrophils (peritoneal cells with surface expression of CD11b^+^/Ly6G^+^) into the peritoneal cavity of WT mice emerged 24 h post‐challenge in response to all three peritonitis insults (**Figure** **7**A). Strikingly, while thioglycollate and zymosan treatments did not reveal significant differences in neutrophil frequency between *Lxrα* ^fl/fl^‐iVav‐Cre^−^ (WT) and *Lxrα* ^fl/fl^‐iVav‐Cre^+^ (Figure 7A,B), a marked reduction in neutrophils was observed in peritoneal exudates from *Lxrα* ^fl/fl^‐iVav‐Cre^+^ mice that were challenged specifically with LPS. These results suggest that endogenous macrophage LXRα plays an unexpected role in controlling the infiltration of immune cells that are recruited to sites of inflammation in response to specific microbial stimuli, such as LPS.

**Figure 8:**
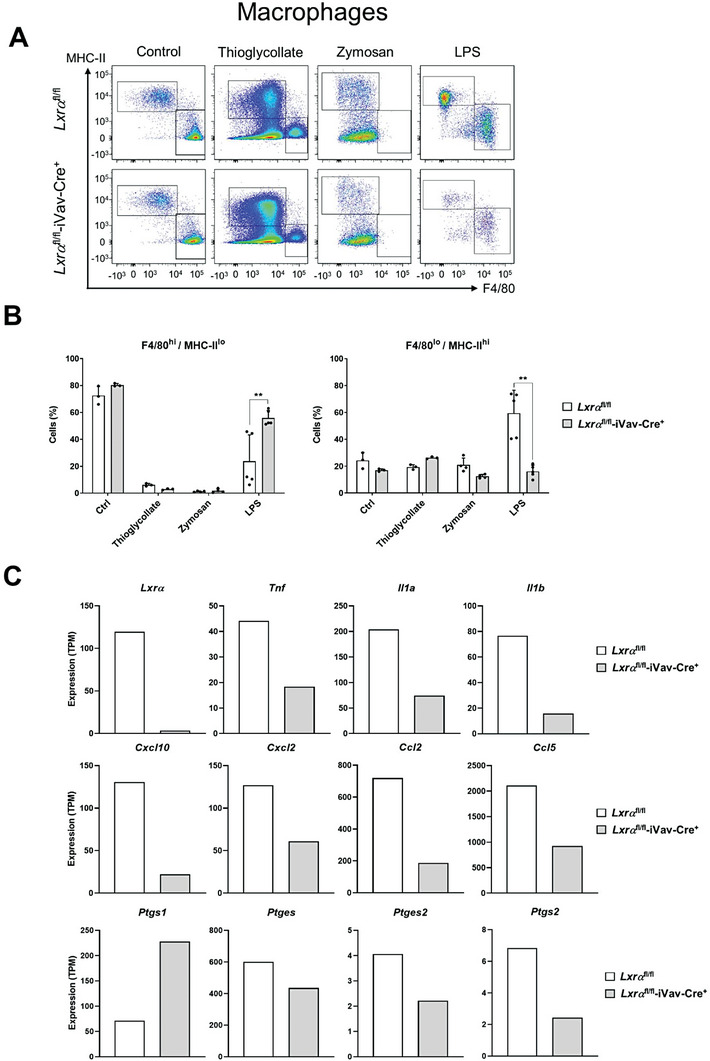
A) Flow cytometry analysis of peritoneal exudates showing subpopulations of peritoneal macrophages in *Lxrα* ^fl/fl^‐iVav‐Cre^−^ (WT) and *Lxrα* ^fl/fl^‐iVav‐Cre^+^ mice, identified as shown in Supporting Figure 7, in control mice and in response to peritonitis stimuli: LPS (4 mg kg^−1^ per mouse), thioglycollate (2 ml of 3% solution per mouse) and zymosan (1 mg per mouse). B) Quantification of flow cytometry data showing the percentage of each macrophage subpopulation among total peritoneal cells. Flow cytometry data were represented as mean ± SD from one or two experiments with n = 4‐5 per group. Significant differences between mean values are denoted with asterisks (** *p* < 0.01). C) mRNA data obtained from FACS‐sorted F4/80^lo^/MHC‐II^hi^ inflammatory macrophages purified from mice that were challenged with LPS for 24 h. Purified cells were obtained from pools of *n* = 4 mice per genotype. Expression values obtained from bioinformatic analysis of RNA‐seq data were represented as Transcripts Per Million reads (TPM).

To explore in depth a possible differential macrophage activation in vivo in the peritoneum that might explain the distinct responses to peritonitis models in the absence of *Lxrα*, we analyzed the frequency and the transcriptional phenotypes of peritoneal macrophages. Under steady‐state conditions, the majority of resident peritoneal macrophages (also called large peritoneal macrophages) express high levels of CD11b and F4/80, but low MHC‐II (designated here as F4/80^hi^/MHC‐II^lo^).^[^ ^47^ ^]^ The second subset, expresses low levels of F4/80 but expresses high levels of MHC‐II, designated here F4/80^lo^/MHC‐II^hi^ (also referred to as small peritoneal macrophages).^[^ ^46^, ^47^ ^]^ First, to examine the role of myeloid LXRα in the differentiation and maintenance of subsets of peritoneal macrophages, we performed flow cytometry analysis of resting peritoneal macrophages from *Lxrα* ^fl/fl^‐iVav‐Cre^−^ (WT) and *Lxrα* ^fl/fl^‐iVav‐Cre^+^ (gating strategy, Figure S7, Supporting Information). Mice deficient in LXRα in hematopoietic cells do not show major differences in the frequency of peritoneal F4/80^hi^/MHC‐II^lo^ or F4/80^lo^/MHC‐II^hi^ macrophage subsets (Figure S7, Supporting Information). In response to inflammatory peritonitis models, the proportion of F4/80^hi^/MHC‐II^lo^ population declines significantly after the challenges, consistent with a previously described reaction known as the “macrophage disappearance reaction”.^[^ ^47^, ^50^ ^]^ The reduction in F4/80^hi^/MHC‐II^lo^ population in response to thioglycollate and zymosan was similar in *Lxrα* ^fl/fl^‐iVav‐Cre^−^ (WT) and *Lxrα* ^fl/fl^‐iVav‐Cre^+^ mice (**Figure** **8**A,B). Remarkably, however, we observed differences in the profile of peritoneal macrophages when mice were challenged with LPS. The proportion of F4/80^lo^/MHC‐II^hi^ macrophage subset increased with LPS in *Lxrα* ^fl/fl^‐iVav‐Cre^−^ (WT) but not in *Lxrα* ^fl/fl^‐iVav‐Cre^+^ mice (Figure 8A,B). Therefore, in the absence of LXR*α*, inflammatory F4/80^lo^/MHC‐II^hi^ macrophages did not expand in the peritoneal cavity to the same extent as in WT mice in response to 24 h of LPS, which may have an impact in the inflammatory mediators expressed by macrophages and the differential recruitment of neutrophils observed in the absence of LXR*α*. This result is consistent with the hypothesis that *Lxrα*‐/‐ macrophages, in an in vivo context, do not acquire the appropriate inflammatory phenotype in response to LPS compared to WT cells.

We further investigated whether the deficit in frequency observed in the inflammatory F4/80^lo^/MHC‐II^hi^ macrophage subset in the absence of LXR*α* would phenocopy some of the characteristics that were observed in vitro, and might present a differential expression of genes involved in inflammation and chemotaxis. We then purified F4/80^lo^/MHC‐II^hi^ macrophages from *Lxrα* ^fl/fl^‐iVav‐Cre^−^ (WT) and *Lxrα* ^fl/fl^‐iVav‐Cre^+^ mice in response to LPS challenge by FACS sorting, and analyze their gene expression by RNA‐seq (Figure 8C). Analysis of differentially expressed genes identified *Tnf, Il1a, Il1b, Ccl2, Ccl5 and Cxcl10, Ptges and Ptges2* whose expression was higher in LPS‐activated *Lxrα* ^fl/fl^‐iVav‐Cre^−^ WT macrophages compared to *Lxrα* ^fl/fl^‐iVav‐Cre^+^ cells. These inflammatory genes encode for chemokines, cytokines or cyclooxygenases and prostaglandin synthases which are known to mediate the infiltration of inflammatory cells into sites of injury. These results support the conclusions obtained in vitro with BMDM and emphasize the importance of LXRα expression in macrophages to acquire a competent expansion and phenotypic activation in vivo in response to bacterial LPS to support the maintenance of secondary inflammatory responses. Together, the results obtained in mice with the peritonitis models identified LXRα as a principal factor in macrophages that sense and amplify the inflammatory response triggered by microbial ligands in vivo.

## 3 Discussion

Macrophages rapidly detect infections and trigger sequential waves of anti‐microbial responses that affect the expression of hundreds of genes.^[^ ^10^, ^51^ ^]^ To facilitate induction of gene expression, chromatin organization must be transformed from repressed basal conditions to allow the binding of SDTFs that drive inflammation‐dependent transcription.^[^ ^52^ ^]^ While the role of pre‐existing transcription factors such as NF‐κB, AP‐1 or IRF‐3 has been extensively studied in macrophages during the initial activation phase,^[^ ^6^, ^42^, ^52^, ^53^ ^]^ less is known about factors that are transcriptionally induced at later phases of inflammatory activation. The studies presented here unveil a previously unrecognized, cell‐autonomous role for LXRα in macrophage inflammatory activation. Induction of LXRα transcription is an important component of macrophage secondary responses that amplify initial inflammation. NF‐κB, IRF‐3 and type I IFNs cooperate in LXRα transcriptional induction, which in turn, facilitates the extension of inflammatory and anti‐microbial responses. We show that endogenous LXRα activity sustains the expression of cytokines and chemokines during inflammation and enables immune‐cell recruitment and activation in response to endotoxin in vivo. Our studies also demonstrate that endogenous LXRα positively regulates secondary inflammatory gene expression through direct binding to cis‐regulatory regions of target genes. This facilitates the recruitment of the p65 component of NF‐κB and possibly other inflammatory SDTFs, that cooperatively promote histone H3K27 acetylation and transcriptional activation. The ability of endogenous LXRα to positively modulate inflammatory gene expression at later stages of anti‐microbial responses provides a plausible mechanism by which endogenous LXRα contributes to innate immune functions.

The role of LXRs in inflammation and host immunity has been the subject of intense investigation since the original studies by some of the authors (AC, PT).^[^ ^16^, ^17^, ^54^ ^]^ Several reports support the major conclusions that ligand‐activated LXRs reciprocally regulate sterol metabolism and inflammation and LXRα/LXRβ dual synthetic agonists have been effectively employed in models of inflammation.^[^ ^55^, ^56^, ^57^ ^]^ In addition, LXR activity, primarily through LXRα, supports innate immunity in murine models of infection,^[^ ^22^, ^23^, ^24^ ^]^ but pretreatment with synthetic LXR ligands may not be beneficial for host immunity as they dampen anti‐microbial inflammatory cascades.^[^ ^20^ ^]^ Thus, the anti‐inflammatory activity of LXR ligands can be dissociated from the innate immune functions driven by endogenous LXR signaling. Our present studies shed light on the function of LXRα in macrophages exposed to microbial ligands in the absence of synthetic LXR agonist supplementation. How can we reconcile the literature and our past studies presenting LXR agonists as anti‐inflammatory agents with our current work that shows endogenous LXRα expression sustaining inflammation? First, the anti‐inflammatory actions of LXR ligands have been well‐documented when agonists are administered hours/days before the insults as preventive therapy.^[^ ^16^, ^17^, ^58^ ^]^ Second, studies demonstrated that repression of inflammation by LXR ligands is dependent on transcriptional induction of ABCA1,^[^ ^50^, ^59^, ^60^ ^]^ which in turn is expressed at membrane lipid microdomains, uncoupling TLR signaling and downregulating NF‐κB and MAPK activation.^[^ ^58^, ^61^ ^]^ Indeed, macrophage‐specific ABCA1 deficient‐mice display increased inflammation and enhanced ability to fight infection.^[^ ^59^ ^]^ In addition, LXR‐dependent expression of other targets, including *Lpcat3*, *Mertk*, *Arginase‐I* and II, also modulate inflammation in response to LXR ligands in different settings.^[^ ^62^, ^63^, ^64^ ^]^ Thus, pharmacological induction of LXR targets by pre‐treatment with synthetic agonists limits subsequent inflammation.

Prior studies revealed the importance of LXR‐dependent gene expression and LXR synthetic ligands as anti‐inflammatory agents.^[^ ^13^ ^]^ However, during macrophage physiological activation, endogenous LXR activity can be regulated by changes in receptor expression and the availability of sterol‐derived ligands,^[^ ^14^, ^15^ ^]^ processes that are both differentially regulated during inflammation. Indeed, cholesterol biosynthetic intermediates, including lanosterol and desmosterol, or oxysterols such 25HC have emerged as potent immune regulators of macrophage activation.^[^ ^65^, ^66^, ^67^, ^68^, ^69^ ^]^ The expression of genes encoding for enzymes of the cholesterol biosynthetic pathway is generally repressed by TLR activation and type I interferons, a response that has been reported to limit lipid availability for invading pathogens and prime cells for improved anti‐microbial immunity.^[^ ^65^, ^70^, ^71^, ^72^, ^73^ ^]^ A notable exception is the Ch25H, whose expression in macrophages is robustly induced (Figure 1D) in a type I interferon‐dependent manner.^[^ ^68^ ^]^ Induction of 25HC production exerts potent anti‐viral actions and appears to stimulate immune activation.^[^ ^67^, ^68^, ^74^ ^]^

In addition, the accumulation of sterol intermediates during macrophage inflammation or foam‐cell formation has been reported to exert different responses, including anti‐inflammatory and anti‐microbial, which have shown different degrees of LXR‐dependency.^[^ ^65^, ^67^, ^75^ ^]^ Our study adds new knowledge about LXRα expression, its epigenomic regulation and function during inflammation. Our analysis of RNA‐seq data from *Ch25h*‐/‐ macrophages showed that some of the inflammatory genes identified as LXRα targets in response to LPS were also dependent on CH25H expression. Thus, changes in levels of various intermediates in the cholesterol biosynthesis or post‐cholesterol oxysterols may have LXR‐dependent and independent outcomes under different temporal dynamics of macrophage activation. Alternatively, we recently described an LXR transcriptional mode of action that is pharmacologically insensitive to agonist modulation,^[^ ^25^ ^]^ suggesting that LXR could additionally function in an LXR ligand‐independent manner under different macrophage pathophysiological settings. In this work, our gene expression analysis adds that, while LXRα transcription is induced to sustain TLR‐dependent macrophage activation, ABCA1 mRNA and protein, and several other LXR targets did not follow such rate of expression. Considering previous literature and new evidence presented here, we propose that TLR‐dependent temporal stimulation of LXRα expression, along with antagonism of classic LXR targets (that limit their anti‐inflammatory activity), favors a greater magnitude of inflammation during prolonged macrophage response to microbial pathogens with a direct implication of LXRα.

Recent studies have documented the genomic landscape of both LXRα/LXRβ in thioglycollate‐elicited peritoneal macrophages or Kupffer cells using ChIP‐seq and dual LXRα,β antibodies,^[^ ^76^, ^77^, ^78^ ^]^ but the individual LXRα and LXRβ genomic binding profiles under different macrophage conditions are still poorly defined. While many LXR transcriptional functions are redundantly performed by LXRα or LXRβ in cultured macrophages in response to synthetic agonists, a unique role of LXRα in the differentiation of liver and spleen resident macrophages has emerged.^[^ ^48^, ^77^, ^78^ ^]^ LXRα is highly expressed in several tissue macrophage subtypes and drives the expression of myeloid genes in response to in vivo‐derived ligands. This suggests that signals and pathways that induce LXRα expression and promote the production of sterol derivatives in local microenvironments may have distinctive pathophysiological implications.^[^ ^69^ ^]^ It will be important for future investigations to study LXRα transcriptional and epigenomic properties in tissue‐resident macrophages under homeostatic or pathological scenarios. Our present contribution, using BMDM that express comparable levels of both LXRs under basal conditions,^[^ ^16^ ^]^ describes a specific mechanism by which TLR‐induced LXRα directly binds to regulatory regions of inflammatory genes and stimulates their expression. In resting conditions, LXRα genomic binding sites are mainly found within the vicinity of genes involved in sterol and fatty acid metabolism. In response to TLR activation, NF‐κB, IRF‐3 and STAT‐1 coordinately facilitate new LXRα transcription. Intriguingly, while the total LXRα protein increased robustly after LPS stimulation, binding of LXRα did not change considerably in LXR classic target‐loci but instead appears notably recruited to regulatory regions of inflammatory genes. Our motif enrichment analysis indicates that newly‐synthesized LXRα appears within predetermined, or ´poised´, IRF‐8/PU.1+ genomic loci. The observation that transcripts induced by LPS in WT macrophages through these collaborative SDTFs along with LXRα binding, but whose expression was attenuated in LXRα‐/‐ macrophages, strongly suggests a direct role for LXRα in their positive regulation during inflammation. Recruitment of LXRα to inflammatory genes facilitates the maintenance of p65 component of NF‐κB at prolonged times after LPS stimulation, presumably in cooperation with other inflammatory SDTFs including IRF‐1 and IRF‐3. Consistent with these results, regions with higher H3K27ac in WT macrophages, associated with active enhancers, were repressed in many inflammatory genes and presented less binding of p65 NF‐κB in LPS‐stimulated *Lxrα*‐/‐ macrophages, suggesting that *Lxrα* expression favors the permanency of NF‐κB and histone acetyl‐transferase complexes within those enhancers during secondary inflammatory responses. The LXRα‐regulated transcriptional activation mechanisms uncovered in cultured macrophages were also validated in vivo with peritonitis models. Studies on the transcriptional components that are involved in macrophage inflammatory response established a coordinated collaboration between different SDTFs that bind to genomic cis‐regulatory regions.^[^ ^5^, ^6^, ^42^ ^]^ Our work on LXRα extends the repertoire of inflammatory SDTFs that collaborate in macrophage secondary inflammatory response and highlights the importance of LXR signaling in host immunity against pathogens. In summary, our findings position LXRα activity at center stage of transcriptional regulation of inflammation and provide a plausible mechanism by which endogenous LXRα contributes to anti‐microbial responses.

## 4 Experimental Section

### Animals and In Vivo Procedures

Mice were maintained under pathogen‐free conditions in a temperature‐controlled room and a 12 h light/dark cycle. Chow and water were available ad libitum. Mouse mutant lines include: C57/BL6 background wild‐type (WT), LXRα‐KO (*Lxrα*−/−), LXRβ‐KO (*Lxrβ*−/−) and LXR‐DKO (*Lxrαβ*−/−) originally obtained from David Mangelsdorf (UTSW).^[^ ^17^ ^]^ Mice bearing a floxed allele of LXRα (*Lxrα* ^fl/+^) on a C57Bl/6 background were obtained from Institut Clinique de la Souris (Illkirch, France); transgenic C57Bl/6 *iVav*‐Cre mice were original from D. Kioussis (NIMC, UK); *Nr1h3* ^fl/+^ and i*Vav*‐Cre+ mice were crossed and resulted in *Lxrα* ^fl/fl^‐i*Vav*‐Cre^+^ with LXRα hematopoietic deficiency that showed macrophage deficiency in vitro and in vivo^[^ ^48^ ^]^ or *Lxrα* ^fl/fl^‐i*Vav*‐Cre^−^ controls. *Irf3* ‐/‐ and *Tnf*‐/‐ bone‐marrow was kindly provided by Dr. Genhong Cheng (UCLA, USA); *Irf1* ‐/‐ was from Dr. Lionel Apetoh (University of Bourgogne, France); *Tlr4*‐/‐ was from Ignacio Lizasoaín (UCM, Madrid); *Stat1* ‐/‐ was from Dr. Ana Planas (IDIBAPS, Barcelona, Spain); *Ifnar1* ‐/‐ and *Myd88* ‐/‐ mice were from Dr. Carlos Ardavín (CNB CSIC, Madrid, Spain); *Trif* ‐/‐ from Dr. Gloria González (CIMA, Universidad de Navarra, Spain). For in vivo peritonitis models, *Lxrα* ^fl/fl^‐i*Vav*‐Cre^+^ mice and *Lxrα* ^fl/fl^‐i*Vav*‐Cre^−^ (4‐5 mice per group) were obtained as littermates and were injected intraperitoneally with either LPS (4 mg kg^−1^ sublethal dose), thioglycollate broth (2 ml of 3% solution as model of sterile peritonitis and potent neutrophil recruitment),^[^ ^24^ ^]^ or with Zymosan challenge at 40 mg kg^−1^. Twenty‐four hours after challenges, animals were sacrificed under isofluorane anesthesia and peritoneal cells were collected by washing with PBS. Cells were pelleted, washed in FACS buffer and prepared for flow cytometry analysis using a FACS Canto II cytometer and FlowJo software. A cohort of mice was employed for the analysis of neutrophil accumulation and a separate cohort used for peritoneal macrophage isolation. Briefly, content of peritoneal lavage from LPS‐challenged mice was subjected to flow cytometry analysis and FACS sorting, according to the gating strategy depicted in Figure S7 (Supporting Information), using CD11b+ pre‐gate and distinction of F4/80^hi^/MHC‐II^lo^ F4/80^lo^/MHC‐II^hi^ macrophages from *Lxrα* ^fl/fl^‐iVav‐Cre^−^ (WT) and *Lxrα* ^fl/fl^‐iVav‐Cre^+^ mice. A fraction of cells was used for flow cytometry analysis (FACS Canto II cytometer) and the rest for FACS‐sorting (MoFlo Astrios sorter). F4/80^lo^/MHC‐II^hi^ macrophages from n = 4 mice per genotype were pooled for RNA analysis. All animal procedures were approved by Institutional Ethic animal Care commissions: Bioethical Commission from Consejo Superior de Investigaciones Científicas PROEX171/18 and ULPGC OEBA‐ULPGC 47/2020.

### Reagents and Antibodies

The following antibodies and conjugates were used in this study: anti‐F4/80 clone Cl:A3‐1. LXRα/β antiserum,^[^ ^43^ ^]^ LXRα (Abcam#PPZ0412), H3K27Ac (Abcam#ab4729), NOS‐2 (Santa Cruz#SC‐650 M‐19), ABCA1 (Novus Biologicals #NB400‐105), GAPDH (Abcam #ab9485‐100), Anti‐mouse‐HRP (Santa Cruz#SC‐2005), Anti‐rabbit‐HRP (Santa Cruz#SC‐2004), CD11b‐PerCP‐Cy5.5 (Biolegend #clone M1/70), F4/80‐PE or FITC (Ebioscience #clone BM8), Ly6G‐PE or FITC (BD Pharmingen #clone AL21), MHC‐II‐APC (eBioscience #M5/114.15.2). The following pharmacological reagents were obtained from the MRC PPU Reagents University of Dundee, UK: BI605906 (IKKβ inhibitor), 5Z‐7‐oxozeanol (TAK1 inhibitor), MRT67307 (TBK1 inhibitor) and the following products from Calbiochem: PD0325901 (MEK/ERK inhibitor), PD98059 (MEK1 and MEK2 inhibitor), PIK‐75‐hydrochloride (inhibitor of p110α subunit of PI3‐Kinase), SB590885 (Raf‐1 inhibitor); HX531 (RXR inhibitor). TLR agonists, Poly I:C (TLR3 agonist) and ultrapure LPS from E. coli 0111:B4 strain‐ (TLR4 ligand) were obtained from Invivogen and were used at 10 µg ml^−1^ and 100 ng ml^−1^ respectively.

### Cell Culture and Macrophage Differentiation

All cells were cultured in RPMI 1640 medium (Lonza) supplemented with 10% fetal bovine serum (Gibco), penicillin 100 U ml^−1^ (Sigma), and streptomycin 100 µg ml^−1^ (Sigma). For BMDM cell differentiation, bone marrow obtained from femur and tibia of 8‐ to 10‐week‐old WT or mutant mice was cultured in non‐adherent petri dishes for 7 days in RPMI supplemented with 10% L929 conditioned medium containing M‐CSF^[^ ^25^ ^]^ and 1% antibiotics (penicillin and streptomycin, Sigma). Parallel experiments to differentiate BMDM were performed with recombinant M‐CSF (Pepro‐Tech, Germany), using a regimen of differentiation as follows: culture media supplemented with 10 ng ml^−1^ added every 2 days to culture media. Macrophage differentiation and activation with L929 and M‐CSF were comparable.

### RNA and Protein Analysis

Whole‐cell lysis was performed with radioimmunoprecipitation assay buffer^[^ ^25^ ^]^ (RIPA; 10 mM Tris‐HCl, pH 7.5, 150 mM NaCl, 1% Triton X‐100, 0.5% sodium deoxycholate, 0.1% SDS, and protease inhibitors, from Roche). Protein extracts were subjected to SDS‐PAGE and transferred to nitrocellulose or polyvinylidene difluoride (PVDF) membranes (Bio‐Rad). Primary antibodies were described above. Reactive bands were detected by Clarity Western ECL substrate (Bio‐Rad). For RNA studies, total RNA was extracted from cells using TRIZOL Reagent (Invitrogen) following manufacturer guidelines. RNA was dissolved in DEPC‐H2O, and 1 µg of RNA was used for iScript cDNA synthesis (Bio‐Rad). For real‐time quantitative PCR, cDNA was used along with 2X PCR MasterMix (Diagenode) specific primer mix. Primer sequences were displayed in Table S1 (Supporting Information). Fluorescence emission in real‐time and analysis was performed with a CFX thermal cycler (Bio‐Rad). The relative levels of RNA were measured following the ΔΔCT method and individual expression data were normalized to 36B4 expression.

### Chromatin Immunoprecipitation (ChIP) Assay

Cell fixation and cross‐linking were performed as follows: First, sets of 5–7 × 10^6^ macrophages cultured in 100 mm non‐adherent petri dishes were cross‐linked with 2 µM disuccinimidyl glutarate (ThermoFisher Scientific) diluted in PBS for 30 min with constant shaking. Cells were then washed with PBS and incubated with 1% methanol‐free formaldehyde (ThermoFisher Scientific) for 10 min. Cross‐linking was quenched with Glycine of 200 mM (Sigma) for 5 min. Chromatin was obtained with two‐step lysis. Hypotonic buffer for nuclear isolation: 50 mM Tris‐HCl, pH 8, 85 mM KCl, 0.5% NP‐40, with Complete (Roche) protease inhibitor. Next, nuclei were incubated with lysis buffer (50 mM Tris‐HCl, pH 8, 10 mM EDTA, 1% SDS, Complete) and stored at −80 °C. Chromatin was sonicated in a Bioruptor (Diagenode) for 60 min (30″ on/30″ off). This regimen yields 100‐400‐bp fragments. A 10% of the total volume was set aside as input control. Immunoprecipitation was performed with 2.5 µg of anti LXR or anti‐H3K27ac antibodies on a total volume of 2 ml in dilution buffer (10 mM Tris‐HCl, pH 8, 2 mM EDTA, 1% Triton X‐100, 150 mM NaCl, and 5% glycerol). Antibody‐bound complexes were isolated with protein A Dynabeads (Thermo‐Fisher Scientific). Washes were performed with 3 buffers: 20 mM Tris‐HCl, pH 8, 2 mM EDTA, pH 8, 1% Triton X‐100, 0.1% SDS, plus 150 mM (first buffer) or 500 mM NaCl (second buffer), and 10 mM Tris‐HCl, 1% sodium deoxycholate, 1 mM EDTA, pH 8, 1% NP‐40, 250 mM LiCl (third buffer). These washes were followed by 2 washes with TE buffer (10 mM Tris‐HCl, pH 8, 1 mM EDTA, pH 8). To perform reverse cross‐linking of protein‐DNA fragments, samples were incubated for 30 min at 37 °C in 1% SDS, 0.1 M NaHCO3, 10 µl of 5 M NaCl, 6 µg ml^−1^ RNase A 1 h at 55 °C with 400 µg ml^−1^ proteinase K (TaKaRa). DNA purification was performed with a Qiagen QIAquick purification kit, and DNA was eluted in a final volume of 50 µl. A fraction was used to confirm enrichment by real‐time qPCR amplification as reported before.^[^ ^25^, ^79^ ^]^

### ChIP Sequencing and Analysis

ChIP dsDNA was quantified using a Qubit 2.0 fluorometer. To prepare libraries, a minimum of 2 ng of precipitated DNA was obtained from the indicated biological replicates per condition. Libraries were prepared by the Genomics Unit of the Centre de Regulacio Genomica (CRG; Barcelona, Spain) using the NEBNext Ultra DNA library preparation kit for Illumina (number 7370) by following the manufacturer's instructions. Twelve cycles of PCR were done for the final library amplification for all samples. Sequencing was performed at the Genomics Unit of the Center for Genomic Regulation (CRG, Barcelona Spain) using Illumina HiSeq2000 equipment. For ChIP‐seq,^[^ ^79^ ^]^ sequencing data (single‐end 50‐bp reads) obtained from Illumina HiSeq2000 were aligned to the UCSC mm10 genome using bowtie2 aligner (v2.2.9) (60). Each ChIP‐seq experiment was normalized to a total number of 1 × 10^7^ uniquely mapped tags. Aligned read files were visualized with IGV (61) genome browser and analyzed with HOMER software (http://homer.ucsd.edu/homer/) (v4.9).^[^ ^5^ ^]^ LXRα binding peaks in each experiment were identified using HOMER and compared to data obtained from LXR‐DKO samples as a negative control.^[^ ^79^ ^]^ LXR peaks and H3K27Ac regions were clustered and represented as tag densities on a heatmap using SEQminer. Ontology analysis of each LXR peak cluster was performed with the Metascape bioinformatics resource.^[^ ^80^ ^]^ Accession numbers for ChIP‐seq data from this study and from public database (GEO) are detailed below. Genomic regions of interest were scanned for transcription factor binding motifs using public JASPAR motif matrix with DMINDA software.

### Transcriptional Profiling and Biological Pathway Analysis

For the global genomic expression study, RNA was obtained using RNeasy MinElute kit (Qiagen), following the manufacturer's instructions. Biological replicate experiments from BMDMs samples were performed and used for analysis in the Affymetrix platform (Affymetrix Mouse Gene 2.0 ST chip). Microarray was performed at the Genomics Unit of the Parque Científico de Madrid‐Universidad Complutense de Madrid. The Expression Console (EC) software was used to normalize the data and to obtain the expression levels in base 2 logarithmic, and the Transcriptome Analysis Console (TAC) software was used to compare the expression levels between the different samples and to obtain the metadata related to those genes differentially expressed. Only changes in gene expression levels >2 compared to reference conditions were considered and also presented a p‐value of less than 0.05. Heatmap representation was performed using the Bioconductor Complex Heatmap package 2.12,^[^ ^43^ ^]^ an R language package (version 2.14.1, in RStudio interface version 0.97.173). For functional enrichment or Gene Ontology (GO) analysis based on differentially expressed gene clusters, Metascape console tool^[^ ^80^ ^]^ was used. Microarray data used in Figure 1a was obtained from the public database https://www.ebi.ac.uk/biostudies/arrayexpress (ArrayExpress) published previously.^[^ ^30^ ^]^ For global RNA sequencing of inflammatory F4/80^lo^/MHC‐II^hi^ peritoneal macrophages, content of peritoneal lavage from LPS‐challenged mice was subjected to FACS sorting. RNA from pooled samples (n = 4‐5; *Lxrα* ^fl/fl^‐iVav‐Cre^−^ (WT) and *Lxrα* ^fl/fl^‐iVav‐Cre^+^ mice treated with LPS for 24 h) was obtained with RNeasy micro‐kit (Qiagen) according to manufacturer instructions. RNA integrity was checked on an Agilent 2100 Bioanalyzer. Library preparation (ultra‐low input kit) and RNA sequencing was carried out by BGI Genomics (Beijing Genomics Institute, China). Bioinformatics analysis was performed using the web‐based solution software BGI Dr. TOM, an in‐house customized data mining system of the BGI (https://biosys.bgi.com). The software performed the enrichment analysis and differentially expressed genes.

### Statistical Analysis

Data were presented as mean ± standard deviation (SD). All determinations were performed in triplicate or quadruplicate and the data shown are representative results from two or three independent experiments or otherwise indicated. Statistical differences between means were tested using Student's t‐test for two groups. Results were considered statistically significant (*) when p<0.05 and highly significant (**) when p<0.01. The statistical Software GraphPad Prism v8.3.0 was used to perform all statistical analyses.

### Accession Numbers

ChIP Sequencing data reported in this paper was deposited in NCBI GEO with accession number: GSE200922. Also, profiling RNA expression data was deposited in NCBI GEO with accession numbers GSE130586 and GSE254257. Public datasets also used in this study were obtained from NCBI GEO accession numbers GSE67343, GSE31796, GSE67343, GSE72964, GSE33913, GSE56123, GSE58993 and Functional Genomics Data ArrayExpress E‐TABM‐310.

## Conflict of Interest

The authors declare no conflict of interest.

## Author Contributions

J.V.d.R., C.T., Z.H. contributed equally to this work. J.V.d.R., C.T., and A.C. performed project conception and design. J.V.d.R., C.T., Z.H., M.C.O., P.M.R., and A.C. performed data curation, analysis, and experimental investigation. K.R.S., J.M.Z., C.T., S.A., L.B., E.T., P.T., and A.C. performed methodology, conceptualization and provide resources. A.C., and E.T. performed funding acquisition. All authors wrote, reviewed and edited the manuscript.

## Supporting information

Supporting Information

## Data Availability

The data that support the findings of this study are openly available in NCBI GEO GSE datasets at https://www.ncbi.nlm.nih.gov/gds, reference number 200922.
