# Supplementary material for: Reprogramming of the LXRα Transcriptome Sustains Macrophage Secondary Inflammatory Responses
Source: Adv Sci (Weinh). 2024 Mar 28;11(20):2307201. doi: 10.1002/advs.202307201 (PMC11132038; doi:10.1002/advs.202307201)
Supplement: Supplementary file 1 — Supporting Information [file ADVS-11-2307201-s001.pdf]

ADVANCED SCIENCE
Open Access

# Supporting Information

for *Adv. Sci.*, DOI 10.1002/advs.202307201

Reprogramming of the LXRα Transcriptome Sustains Macrophage Secondary Inflammatory Responses

*Juan Vladimir de la Rosa, Carlos Tabraue, Zhiqiang Huang, Marta C. Orizaola, Patricia Martin-Rodríguez, Knut R. Steffensen, Juan Manuel Zapata, Lisardo Boscá, Peter Tontonoz, Susana Alemany, Eckardt Treuter and Antonio Castrillo**

# FIGURE S1

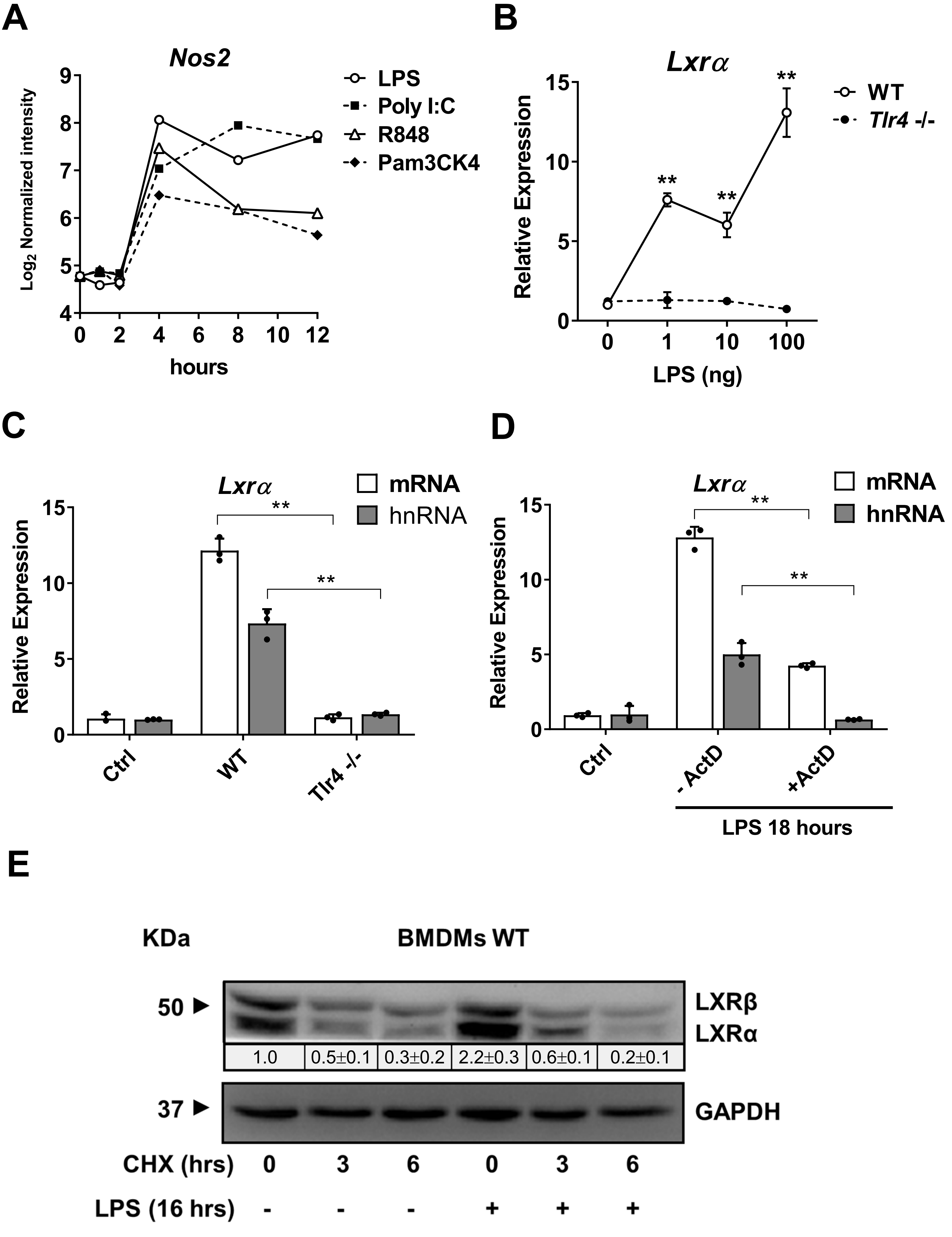
A
Nos2
Log2 Normalized intensity
hours
LPS
Poly I:C
R848
Pam3CK4
B
Lxrα
Relative Expression
LPS (ng)
WT
Tlr4 -/-
C
Lxrα
mRNA
hnRNA
Ctrl
WT
Tlr4 -/-
D
Lxrα
- ActD
+ActD
LPS 18 hours
E
KDa
BMDMs WT
50
37
LXRβ
LXRα
GAPDH
1.0
0.5±0.1
0.3±0.2
2.2±0.3
0.6±0.1
0.2±0.1
CHX (hrs)
LPS (16 hrs)

**Figure S1.**

**(A)** Relative *Nos2* mRNA expression in BMDM cultured with TLR agonists: LPS (TLR4), Poly I:C (TLR3), PAM3CSK4 (TLR2) and R848 (TLR7 and TLR8); normalized values of RNA expression were obtained from database ArrayExpress E-TABM-310[30]. **(B)** Relative *Lxrα* mRNA levels in WT and *Tlr4*-/- BMDMs cultured with LPS (1-100 ng $ml^{-1}$) for 24 hours. **(C)** Relative *Lxrα* mRNA and nascent hnRNA in WT and *Tlr4*-/- BMDMs cultured with LPS (1-100 ng $ml^{-1}$) for 24 hours (left panel). **(D)** Relative *Lxrα* mRNA and nascent hnRNA in WT BMDM cultured with or without LPS (100 ng $ml^{-1}$) for 18 hours and then challenged with Actinomycin D (10ug $ml^{-1}$) for 6 hours (panel on the right). **(E)** Expression levels of LXRα, LXRβ and GAPDH proteins in BMDM control or LPS stimulated for 16 hours and then either untreated or cultured with Cycloheximide (CHX, 10ug $ml^{-1}$) for 3 or 6 hours as indicated. Levels of LXRα protein were quantified by densitometry of band intensity. mRNA or nascent hnRNA expression data were represented as mean ± SD from 3 experiments. Significant differences between mean values of mRNA or nascent hnRNA are denoted (** $p < 0.01$)

# FIGURE S2

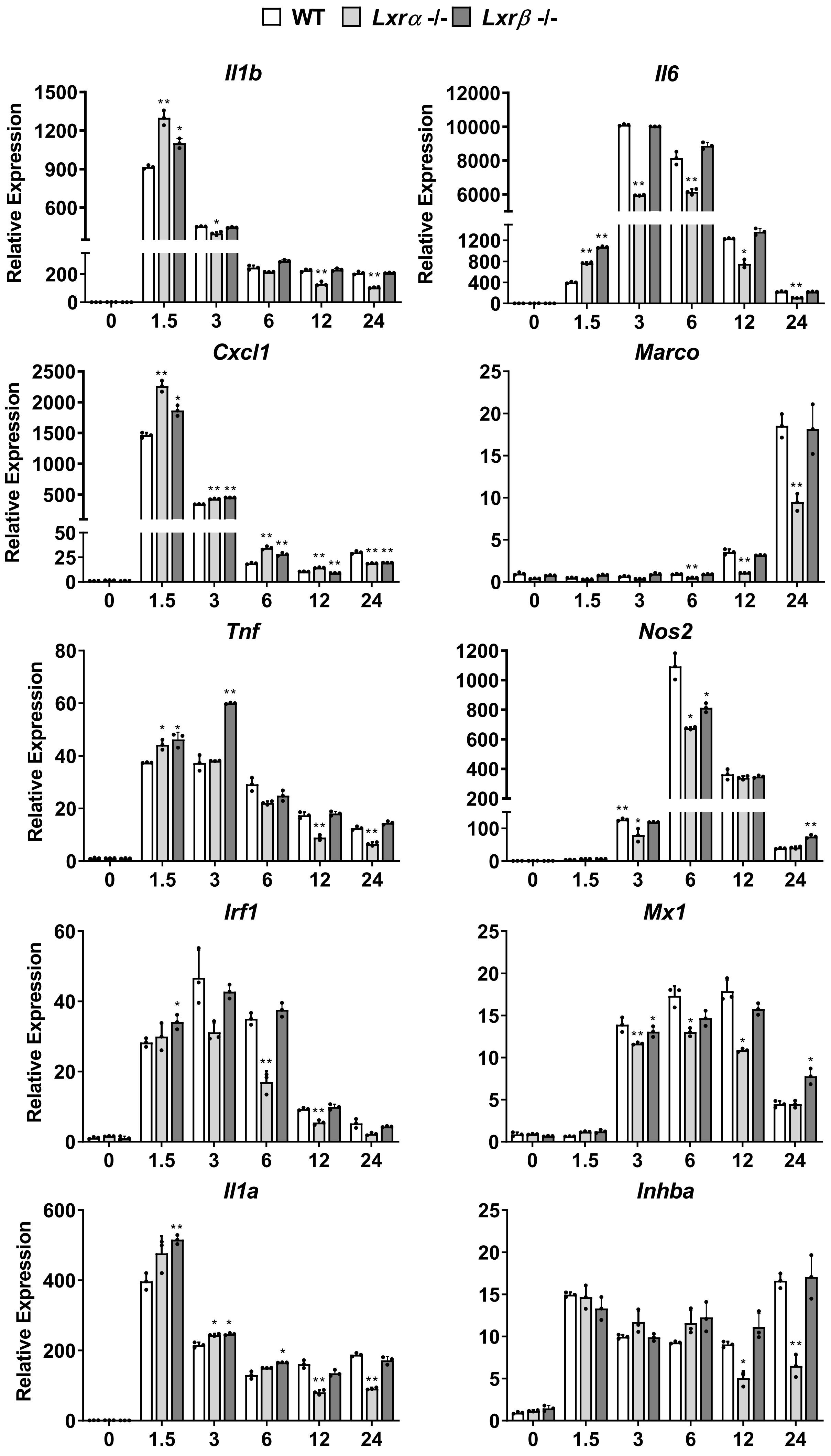

WT
Lxrα -/-
Lxrβ -/-
Il1b
Il6
Cxcl1
Marco
Tnf
Nos2
Irf1
Mx1
Il1a
Inhba
Relative Expression
0
1.5
3
6
12
24

**Figure S2.**

Time-course of mRNA expression of the indicated genes at different times (0, 1.5, 3, 6, 12, 24 h.) in *Lxr*α-/- or *Lxr*β-/- BMDMs cultured with or without LPS (100 ng $ml^{-1}$). Data were represented as mean ± SD from 2 experiments. Significant differences between mean values of *Lxr*α-/- or *Lxr*β-/- cells compared to WT within each time are denoted (** $p < 0.01$, *$p < 0.05$)

# FIGURE S3

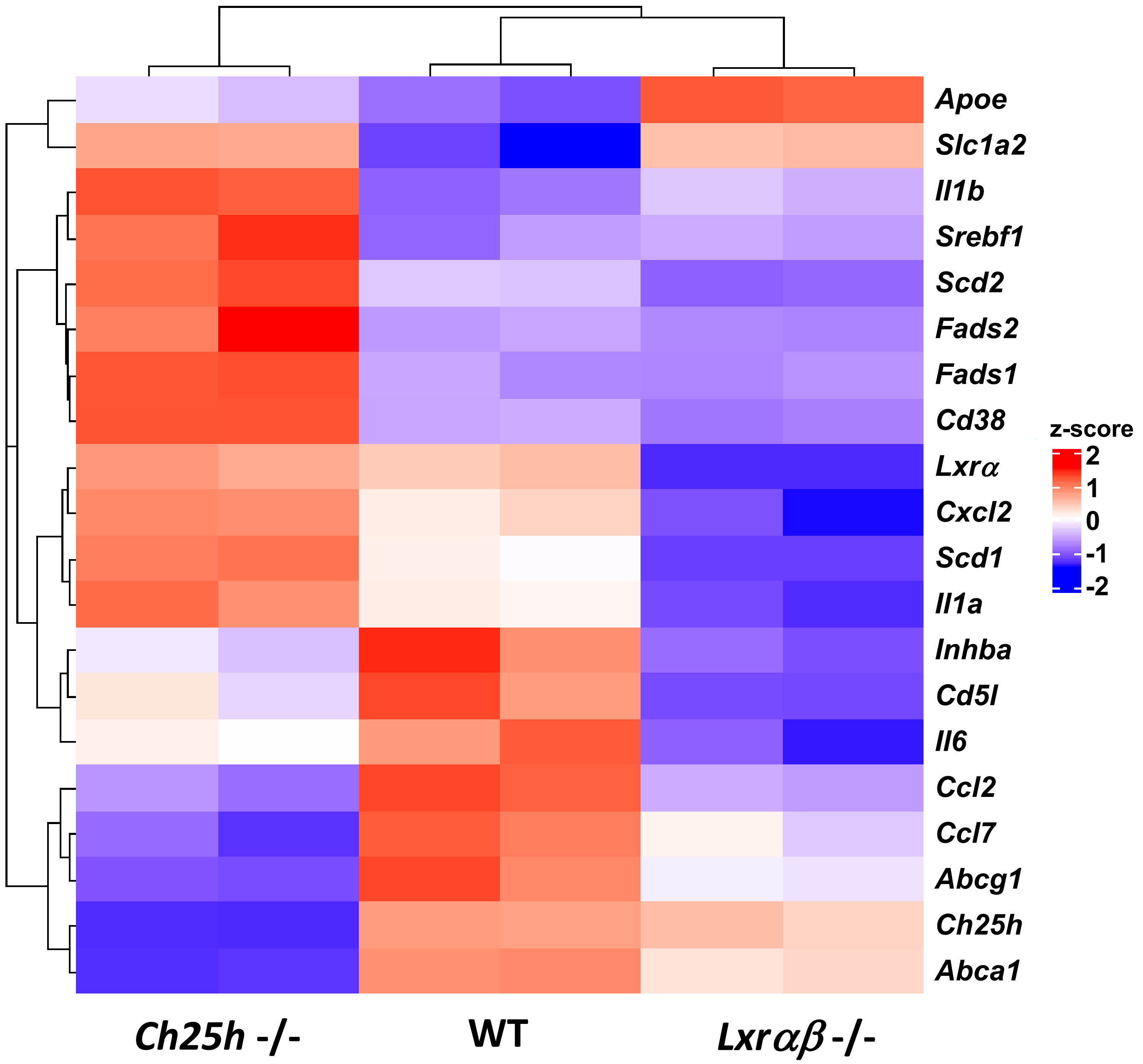
Apoe
Slc1a2
Il1b
Srebf1
Scd2
Fads2
Fads1
Cd38
Lxrα
Cxcl2
Scd1
Il1a
Inhba
Cd5l
Il6
Ccl2
Ccl7
Abcg1
Ch25h
Abca1
z-score
2
1
0
-1
-2
Ch25h -/-
WT
Lxrαβ -/-

**Figure S3.**

Heatmap of mRNA expression of a selected panel of genes in WT and *Lxrαβ*-/- and *Ch25h*-/- BMDMs; data analyzed from public datasets GSE58993. Each row is z-score normalized.

**FIGURE S4**

**A**

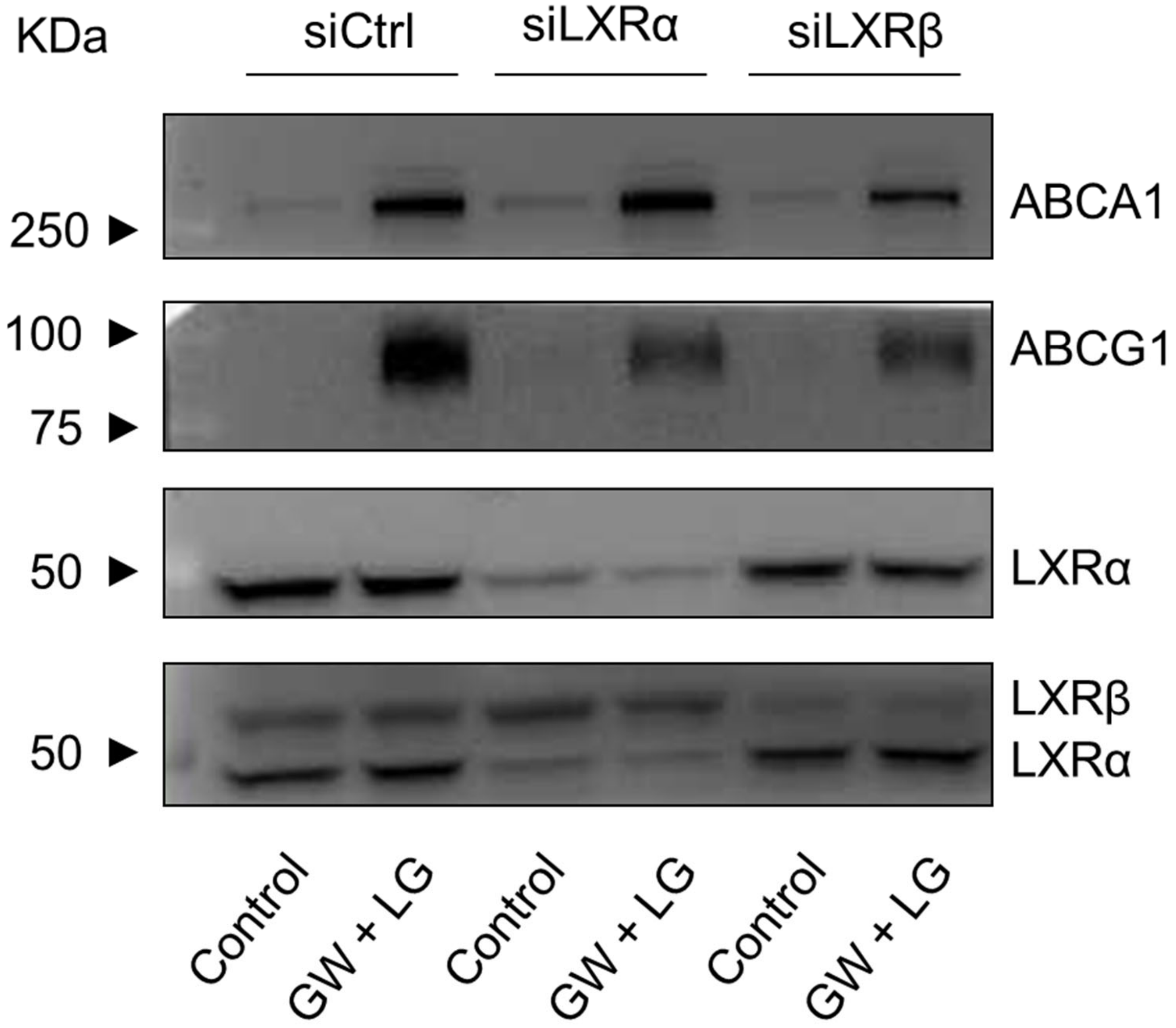
KDa
siCtrl
siLXRα
siLXRβ
250
ABCA1
100
75
ABCG1
50
LXRα
50
LXRβ
LXRα
Control
GW + LG
Control
GW + LG
Control
GW + LG

**Figure S4.**

Protein levels of ABCA1, ABCG1, LXRα or LXRαβ in WT BMDMs transfected with control siRNA or with LXRα or LXRβ specific siRNAs smartpool. Cells were cultured with or without LXR agonists GW3965 (2 μM) and LG268 (100 nM) for 24h.

# FIGURE S5

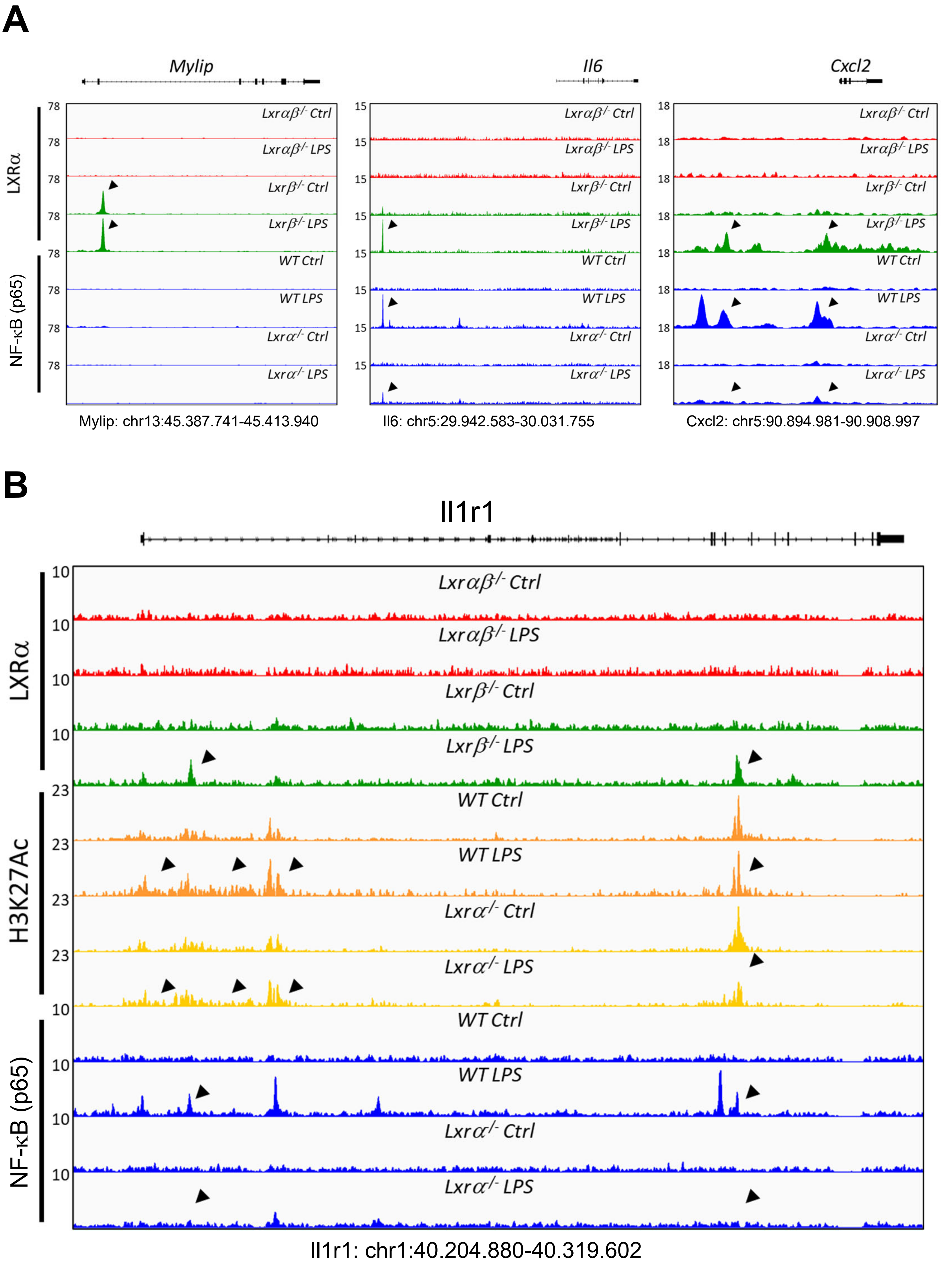

A
Mylip
Il6
Cxcl2
LXRα
NF-κB (p65)
Lxrαβ-/- Ctrl
Lxrαβ-/- LPS
Lxrβ-/- Ctrl
Lxrβ-/- LPS
WT Ctrl
WT LPS
Lxrα-/- Ctrl
Lxrα-/- LPS
78
15
18
Mylip: chr13:45.387.741-45.413.940
Il6: chr5:29.942.583-30.031.755
Cxcl2: chr5:90.894.981-90.908.997
B
Il1r1
LXRα
H3K27Ac
NF-κB (p65)
Lxrαβ-/- Ctrl
Lxrαβ-/- LPS
Lxrβ-/- Ctrl
Lxrβ-/- LPS
WT Ctrl
WT LPS
Lxrα-/- Ctrl
Lxrα-/- LPS
10
23
Il1r1: chr1:40.204.880-40.319.602

**Figure S5.**

**(A)** IGV genome browser images of LXRα ChIP-seq binding sites for the indicated loci of vehicle or LPS-treated *Lxr*β-/- BMDM (LXRα binding in green). As negative control, ChIP-seq in LXRαβ-/- BMDM (in red). In blue, binding of p65 NF-κB ChIP-seq in control and 24h LPS stimulated WT or *Lxr*α-/- BMDM
**(B)** IGV genome browser images of ChIP-seq binding sites for the *Il1r1* locus of vehicle or LPS-treated *Lxr*β-/- BMDM (LXRα in green in LXRβ-/- BMDMs), H3K27ac (orange/yellow in WT or *Lxr*α-/- BMDM) and p65 NF-κB (blue in WT or *Lxr*α-/- BMDM).

# FIGURE S6

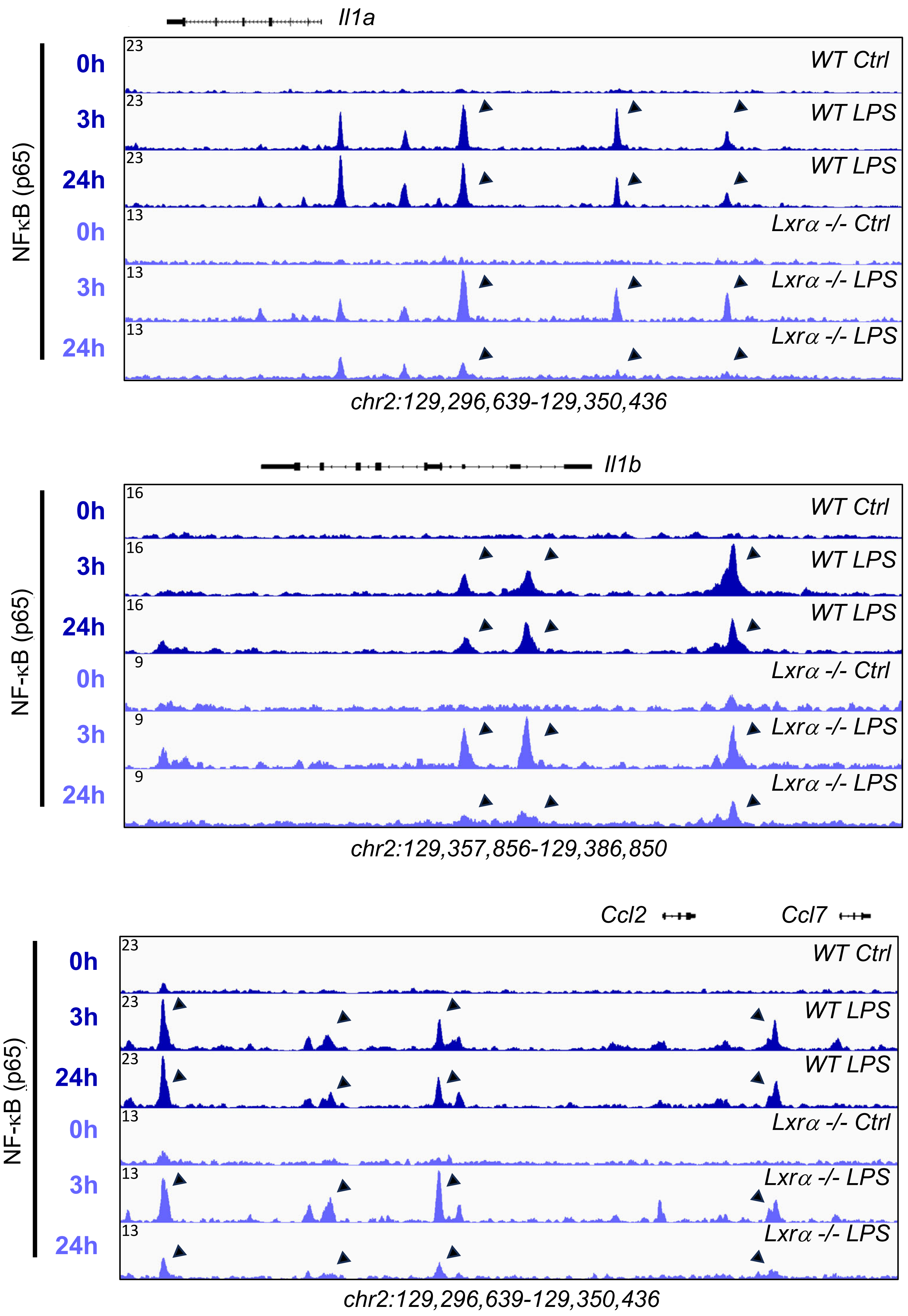

Il1a
NFκB (p65)
0h
3h
24h
0h
3h
24h
23
WT Ctrl
WT LPS
WT LPS
13
Lxrα -/- Ctrl
Lxrα -/- LPS
Lxrα -/- LPS
chr2:129,296,639-129,350,436
Il1b
NF-κB (p65)
16
9
chr2:129,357,856-129,386,850
Ccl2
Ccl7
chr2:129,296,639-129,350,436

**Figure S6.**

IGV genome browser images for the indicated loci (*Il1a, Il1b* and *Ccl2*) of p65 NF-κB ChIP-seq in control, 3h and 24h LPS stimulated in WT or *Lxr*α-/- BMDM.

# FIGURE S7

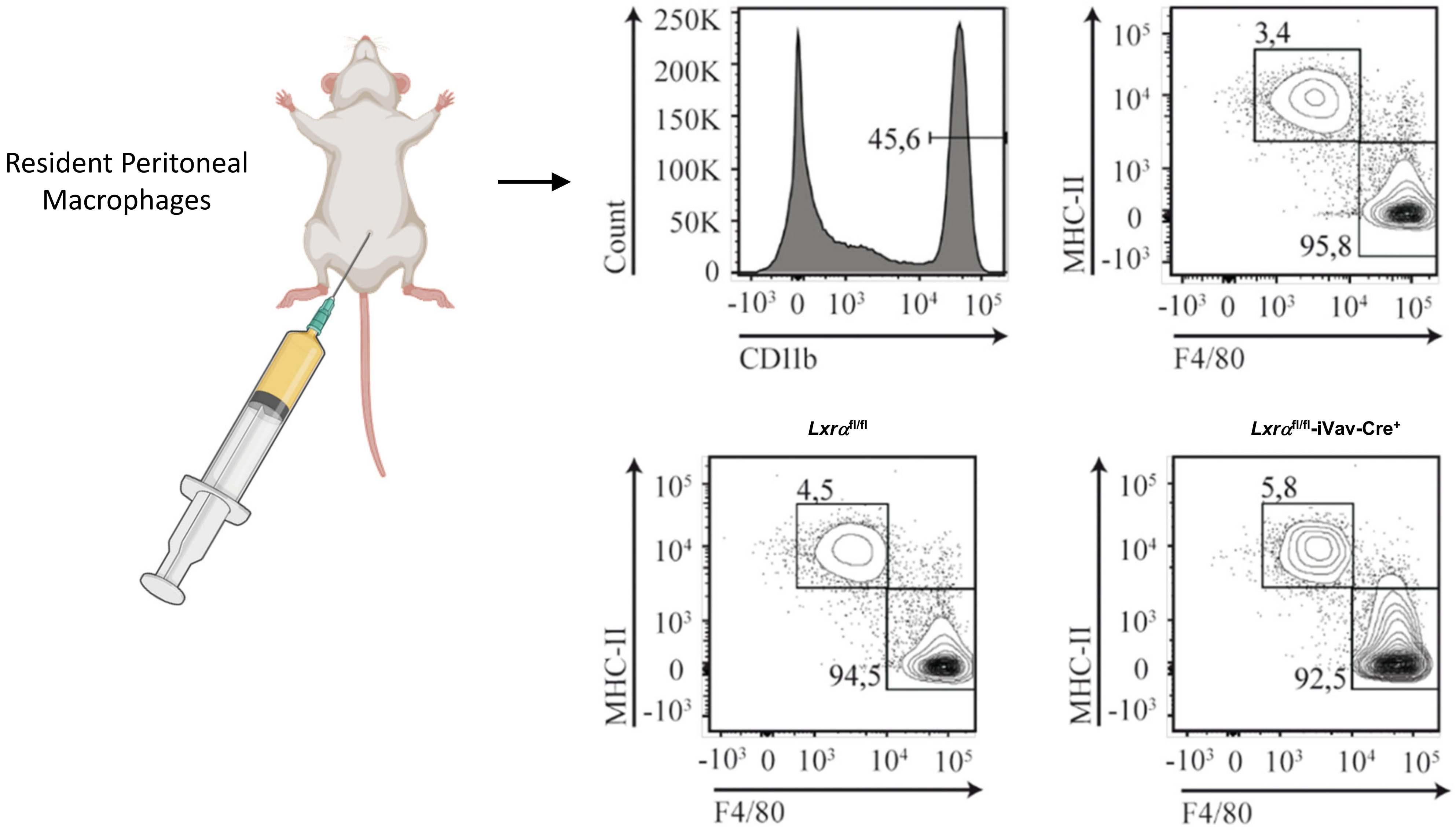

Resident Peritoneal Macrophages
Count
250K
200K
150K
100K
50K
0
45,6
-10³ 0 10³ 10⁴ 10⁵
CD11b
MHC-II
3,4
95,8
F4/80
*Lxrα*fl/fl
4,5
94,5
*Lxrα*fl/fl-iVav-Cre+
5,8
92,5

**Figure S7.**

Gating strategy for the identification of mouse peritoneal macrophages. Peritoneal exudate was obtained by injecting PBS into peritoneal cavity and cells were stained with CD11b, F4/80 and MHC-II antibodies. A representative flow cytometry plot from *Lxrα*$^{fl/fl}$-iVav-Cre$^{-}$ (WT) and *Lxrα*$^{fl/fl}$-iVav-Cre$^{+}$ mice is represented.

TABLE S1. PRIMERS FOR REAL-TIME QPCR

| Transcript | Orientation | Sequence |
|---|---|---|
| ***36b4*** | FWD | 5’- GGC CCT GCA CTC TCG CTT TC -3’ |
| | REV | 5’- TGC CAG GAC GCG CTT GT -3’ |
| ***Abca1*** | FWD | 5'- GCA GAT CAA GCA TCC CAA CT-3’ |
| | REV | 5'- CCA GAG AAT GTT TCA TTG TCC A-3’ |
| ***Abcg1*** | FWD | 5'- TCA CCC AGT TCT GCA TCC TCT T -3’ |
| | REV | 5'- GCA GAT GTG TCA GGA CCG AGT -3’ |
| ***Apoe*** | FWD | 5'- AAC AGA CCC AGC AAA TAC GCC -3’ |
| | REV | 5'- CTC ATT GAT TCT CCT GGG CC -3’ |
| ***Ccl2*** | FWD | 5’- CAT CCA CGT GTT GGC TCA-3’ |
| | REV | 5’- GAT CAT CTT GCT GGT GAA TGA GT-3’ |
| ***Cd38*** | FWD | 5'- AAA ACT TCT CCA TTC CAT CTG TTA CA -3’ |
| | REV | 5'- CAT GTG TGT CCA AGG CAT TAA AC -3’ |
| ***Cd5l*** | FWD | 5'-TTT GTT GGA TCG TGT TTT TCA GA -3' |
| | REV | 5'- CTT CAC AGC GGT GGG CA -3' |
| ***Ch25h*** | FWD | 5’-GCGACGCTACAA GATCCA-3' |
| | REV | 5’-CACGAACACCAGGTGCTG-3’ |
| ***Cxcl1*** | FWD | 5'-ATCCAGAGCTTGAAGGTGTTG-3' |
| | REV | 5'-GTCTGTCTTCTTTCTCCGTTACTT-3' |
| ***Fads1*** | FWD | 5'- TCA GCG ACT TCA GCC -3' |
| | REV | 5'- AAA AGG ATC CGT GGC AT -3' |
| ***Fads2*** | FWD | 5'- AAG GGA GGT AAC CAG GGA GAG -3' |
| | REV | 5'- CCG CTG GGA CCA TTT GGT AA -3' |
| ***Il1a*** | FWD | 5'- TTG GTT AAA TGA CCT GCA ACA -3’ |
| | REV | 5'- GAG CGC TCA CGA ACA GTT G -3’ |
| ***Il1b*** | FWD | 5’-TCT TCT TTG GGT ATT GCT TGG-3’ |
| | REV | 5’-TGT AAT GAA AGA CGG CAC ACC-3’ |
| ***Il6*** | FWD | 5'- CCA GGT AGC TAT GGT ACT CCA GAA -3’ |
| | REV | 5'- GCT ACC AAA CTG GAT ATA ATC AGG A -3’ |
| ***Inhba*** | FWD | 5' - ATC ATC ACC TTT GCC GAG TC - 3' |
| | REV | 5' - TCA CTG CCT TCC TTG GAA AT - 3' |
| ***Irf1*** | FWD | 5'- CTT TGA ACA GTC TGA GTG GCA G -3’ |
| | REV | 5'- CCC ATC AGG AGG TTT CCT CG -3’ |

| | | |
|---|---|---|
| ***Lxrα (hnRNA)*** | FWD | 5'- CAG CTC AGT AAA GTG GGC AA -3' |
| | REV | 5'- TCG ACT CAC AGC ACT TTA CCT -3' |
| ***Lxrα (mRNA)*** | FWD | 5'- CCT TCC TCA AGG ACT TCA GTT ACA T-3' |
| | REV | 5'- CAT GGC TCT GGA GAA CTC AAA GAT-3' |
| ***Lxrβ*** | FWD | 5'- CCC CAC AAG TTC TCT GGA CAC T-3' |
| | REV | 5'- TGA CGT GGC GGA GGT ACT G-3' |
| ***Marco*** | FWD | 5'- GGC ACC AAG GGA GAC AAA -3' |
| | REV | 5'- TCC CTT CAT GCC CAT GTC -3' |
| ***Mx1*** | FWD | 5'- AAA CCT GAT CCG ACT TCA CTT CC -3' |
| | REV | 5'- TGA TCG TCT TCA AGG TTT CCT TGT -3' |
| ***Nos2*** | FWD | 5'- GCA GCT GGG CTG TAC AAA -3' |
| | REV | 5'- AGC GTT TCG GGA TCT GAA T -3' |
| ***Scd1*** | FWD | 5'- TCA GCA CTG GGA AAG TGA GG -3' |
| | REV | 5'- AAC TGG AGA TCT CTT GGA GCA T -3' |
| ***Scd2*** | FWD | 5'- CTG GAA ATG CAA AGA CCG GA -3' |
| | REV | 5'- AGA CGC GGA GAG GTA CAT AA -3' |
| ***Tnf*** | FWD | 5'- ATC ATC TTC TCA AAA TTC GAG TGA -3' |
| | REV | 5'- TTG AGA TCC ATG CCG TTG G -3' |
